# Bridging Conventional and Natural Therapies for Recurrent Aphthous Stomatitis: A Systematic Review and Meta-Analysis of Triamcinolone and Non-Steroidal Alternatives

**DOI:** 10.3390/biomedicines14102260

**Published:** 2026-10-06

**Authors:** Rana A. Elbeshbeishy, Biji Thomas George, Mohamed Anas Patni, Ragai Gemi, Mohamed R. Gemae, Lovely M. Annamma

**Affiliations:** 1 Department of Anatomy, RAK College of Medical Sciences, RAK Medical and Health Sciences University, Ras Al Khaimah 11172, United Arab Emirates; rana.aly@rakmhsu.ac.ae or ranaelbeshbishy@gmail.com; 2 Department of Surgery, RAK College of Medical Sciences, RAK Medical and Health Sciences University, Ras Al Khaimah 11172, United Arab Emirates; biji@rakmhsu.ac.ae; 3 Department of Community Medicine, RAK College of Medical Sciences, RAK Medical and Health Sciences University, Ras Al Khaimah 11172, United Arab Emirates; mohamedanas@rakmhsu.ac.ae; 4 Department of Otolaryngology-Head and Neck Surgery, Al Qassimi Hospital, Sharjah P.O. Box 3500, United Arab Emirates; ragaiegemaie@hotmail.com or ragai.gemi@ehs.gov.ae; 5 Department of Ophthalmology and Vision Sciences, University of Toronto, Toronto, ON M5T 3A9, Canada; mohamed.gemae@mail.utoronto.ca; 6 Department of Clinical Sciences, College of Dentistry, Ajman University, Ajman P.O. Box 346, United Arab Emirates; 7 Adjunct Faculty, Dental Research Cell, Patil Dental College & Hospital, Patil Vidyapeeth, Pune 411018, India

**Keywords:** recurrent aphthous stomatitis, randomized controlled trial, natural herbal, laser therapy, triamcinolone

## Abstract

**Background**: Recurrent aphthous stomatitis (RAS) is a chronic inflammatory condition that causes painful oral ulcers that impair eating and speech. Topical triamcinolone is commonly used, but non-steroidal natural and synthetic alternatives are increasingly studied. This systematic review and meta-analysis compared alternative topical therapies for pain outcomes (VAS, pain recovery time) and ulcer-healing time with topical triamcinolone. **Methods**: A PRISMA-guided search of five electronic databases for randomized controlled trials (RCTs) published between March 2014 and March 2025 was carried out. Eligible studies that compared alternative or natural treatments with topical triamcinolone for RAS based on randomized clinical trials were considered. Data were synthesized with random-effects models using standardized mean differences (SMDs) and 95% confidence intervals (CIs). Random-effects analyses combining different interventions were interpreted as secondary, exploratory syntheses. **Results**: Twenty-six RCTs were included qualitatively; 14 RCTs (*n* = 836) contributed to the pain (VAS) meta-analysis (428 experimental; 408 triamcinolone). Alternative treatments had a similar reduction in pain to triamcinolone (SMD = 0.37, 95% CI 0.04–0.69; *p* < 0.03), with substantial heterogeneity (I^2^ ≈ 80%). Pain recovery time (4 RCTs; *n* = 182) did not differ significantly (SMD = 2.62; *p* = 0.36) and was highly heterogeneous. Ulcer-healing time (5 RCTs; *n* = 245) was shorter with alternatives (SMD = −1.28, 95% CI −2.33 to −0.23; *p* < 0.05). Reported alternatives included natural agents (e.g., honey, sage, pomegranate, curcumin) and synthetic modalities (e.g., hyaluronic acid, mucoadhesive patches, laser therapy). **Conclusions**: Some individual studies reported favourable pain or healing outcomes with alternatives to topical triamcinolone. However, limited treatment-specific evidence and substantial clinical heterogeneity restrict firm conclusions. Pooled estimates across different interventions should be considered exploratory. Further rigorous trials are needed to establish the comparative effectiveness and safety of individual treatments.

## 1. Introduction

Recurrent aphthous stomatitis (RAS) (aphthous stomatitis, canker sores, mouth ulcers and recurrent aphthous ulcers) is a chronic inflammatory condition with recurrent painful ulcers, affecting the oral mucosa involving non-keratinized mucosa such as buccal, labial mucosa, tongue and floor of the mouth [1]. The pathogenesis of RAS is characterized by the disruption of the epithelial layer, accompanied by a local inflammatory response [2]. RAS causes discomfort in eating, speaking and drinking, along with pain, which affects quality of life. The contributing factors include a compromised immune system, stress, nutritional deficiencies, trauma, and genetic predisposition [2]. Recurrent aphthous stomatitis is commonly classified as minor aphthous ulcers, major aphthous ulcers and herpetiform ulcerations [3]. Cell-mediated inflammation in RAS involves T cells and tumour necrosis factor-alpha [4]. Minor ulcers are small (less than 1 cm in diameter) and generally heal within 7–10 days without scarring. Major aphthous ulcers, which are deeper than 1 cm and more painful, take several weeks to heal and often leave scars. Herpetiform aphthous ulcers occur in clusters and are difficult to manage [5,6]. Various treatment modalities, including topical applications, mouthwashes, and tablets, are available and include corticosteroids, analgesics, probiotics, and antibiotics.

The most often used topical corticosteroid medications are triamcinolone and hydrocortisone for the treatment of RAS [7]. The other frequently used medications include analgesics and anti-inflammatories, such as benzocaine, lidocaine, and amlexanox [8,9]. Triamcinolone, a synthetic corticosteroid, works by inhibiting the production of pro-inflammatory cytokines, thereby alleviating pain and reducing ulcer size [10]. While effective, long-term use is associated with potential side effects, including mucosal thinning and delayed healing [11]. Researchers have explored structural modifications to triamcinolone, such as acetonide formation, the addition of labile ester bonds, and polymer conjugation, to influence drug release, tissue exposure, solubility, and local retention. These modifications aim to improve how the corticosteroid is delivered and handled by the body while preserving its anti-inflammatory effect. Plant-derived compounds, including polyphenols, flavonoids, and terpenoids, have been studied for their anti-inflammatory, antioxidant, analgesic, and tissue-healing effects. Their ability to modulate inflammatory processes and support mucosal healing has potential use for managing recurrent aphthous stomatitis. Natural and other topical applications include low-level lasers (diodes and Er: YAG) [12], doxycycline [13], probiotics [14], ozone [15], insulin liposome gel [16], chlorhexidine gluconate (e.g., Peridex) [17], and hyaluronic acid [18]. Natural and herbal remedies include aloe vera [19], chamomile [20], liquorice root (*Glycyrrhiza glabra*) [21], coconut oil [22], honey/bee propolis [23], and camel whey protein [24], *Punica granatum* gel [25] and triadent oral paste [26] are also used for managing aphthous ulcers. Many natural and herbal remedies have been studied to avoid the side effects of corticosteroids. Other alternative treatments that have been reported to be effective include laser therapy [12], ozone [15], and probiotics [14]. While these natural agents offer promising therapeutic options, their effectiveness compared with conventional corticosteroids such as triamcinolone remains a critical area of study. The reason for choosing Triamcinolone as the comparator was that it is an established topical corticosteroid commonly used to manage the symptoms of recurrent aphthous stomatitis. It has also been widely used as a comparator in clinical trials investigating alternative topical treatments. Using triamcinolone as the reference therefore provides a clinically familiar basis for comparing the effects of non-steroidal interventions on pain relief and ulcer healing. This systematic review and meta-analysis aims to compare the efficacy of natural or alternative topical agents with topical triamcinolone. The parameters studied were pain reduction, VAS scores, and ulcer healing time.

## 2. Materials and Methods

The systematic review adhered to the Preferred Reporting Items for Systematic Reviews and Meta-Analyses (PRISMA) (Supplementary S1) [27]. It was registered in PROSPERO (registration number: CRD420250655772). The current systematic review aimed to evaluate the efficacy of the most effective topical treatment for RAS by comparing the commonly used medication, topical triamcinolone, with other natural and alternative topical treatments. Analyses combining different interventions were considered secondary and exploratory.

### 2.1. Data Collection Process

The data comparing efficacy, based on pain relief (VAS), pain recovery time, and ulcer healing, were retrieved from randomized controlled trials (parallel group and split-mouth designs) evaluating topical interventions published between March 2014 to March 2025 (Table 1). For split mouth trials, paired data were analyzed using appropriate within subject comparisons, and effect sizes were calculated accounting for the paired nature of the data. The focus question is as follows:

Do ulcer healing time, pain relief and pain recovery time of other topical natural or alternative remedies compare with topical triamcinolone for the treatment of RAS?

#### 2.1.1. PICO

P, Population of individuals with RAS.

I, Intervention: pharmacological and nonpharmacological topical or alternative treatments for RAS.

C, Comparison of topical triamcinolone with other topical applications or alternative treatments for RAS.

O, Outcome: pain relief, pain recovery time, and overall ulcer healing time.

#### 2.1.2. Inclusion Criteria

RCTs evaluating pharmacological or nonpharmacological topical treatments for RAS.

Studies that reported outcomes such as pain relief, reduction in ulcer size, and reduced healing time.

Studies published in English, with a follow-up duration ranging from one day up to one year.

#### 2.1.3. Exclusion Criteria

Non-randomized studies, studies not focusing on treatments for RAS, case reports, abstracts, or conference proceedings.

Aphthous ulcers with systemic involvement, such as Behcet’s disease, Crohn’s disease, ulcerative colitis, hematologic disorders, immunodeficiency and other systemic inflammatory conditions, were excluded based on confirmed clinical history and diagnostic criteria.

All non-topical applications (systemic) for the treatment of RAS.

All systematic reviews were excluded.

Study population, interventions, and outcome indicators that did not meet the inclusion criteria.

Non-relevant outcomes (e.g., pain levels not directly measured, pain results not specifically reported).

The primary outcome was reduction in pain intensity measured using the Visual Analog scale, and secondary outcomes included pain recovery time and ulcer healing time.

#### 2.1.4. Data Items

The search is compatible with OVID EMBASE, Ovid MEDLINE and the Cochrane Library. Keywords were also used for the Web of Science and Scopus databases.

As the review involved the analysis of previously published data, no ethical approval was required. Five electronic databases were used: Medline, the Cochrane Central Register of Controlled Trials (Central), Embase, Scopus and Web of Science. The MeSH terms used are cited in Table 1.

**Figure 1:**
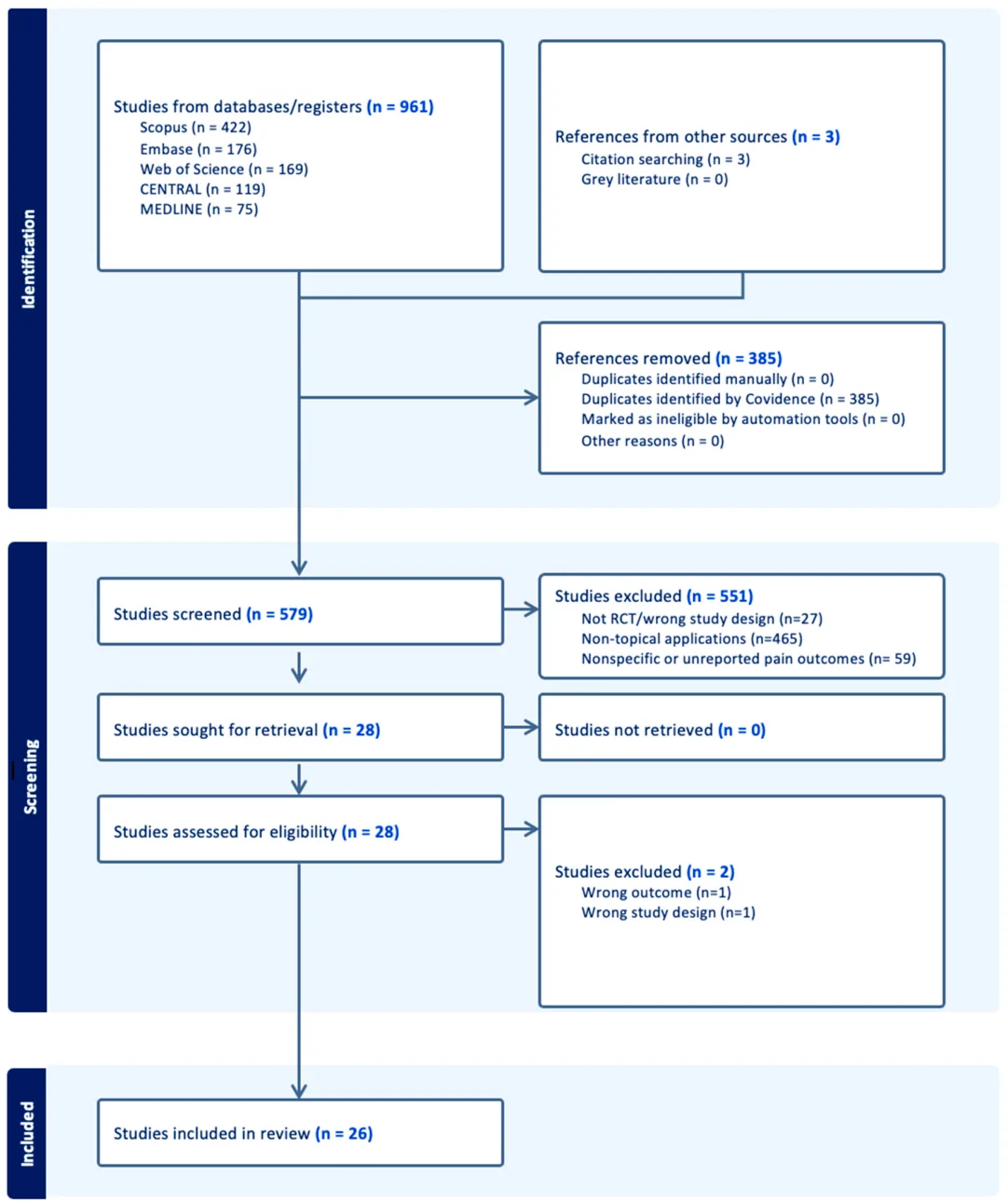
PRISMA chart.

After the initial search, 961 articles were retrieved from the five databases, of which 385 were removed after duplicates were eliminated. The remaining 579 articles were retrieved as abstracts and assessed for eligibility according to the inclusion and exclusion criteria. The remaining 28 studies were selected, and their full texts were retrieved. Two further studies were excluded due to errors in outcomes and study design, leaving 26 studies which were subsequently subjected to data extraction (Figure 1).

Three independent reviewers collected data and reviewed them separately, which included the author(s), year of publication, study location, study design, sample size, intervention type, mean duration of pain recovery, reduction in ulcer size or complete recovery, duration of follow-up, and outcome. A second group of three reviewers resolved any disagreements that arose and any missing data.

#### 2.1.5. Statistical Analysis

Intervention-specific findings formed the primary basis for interpretation. Meta-analysis within an intervention was undertaken only where usable data and sufficient clinical and methodological compatibility were available; otherwise, individual-study findings were presented descriptively. Standardized mean differences (SMDs) with 95% confidence intervals (CIs) were used for quantitative synthesis. Analyses combining different interventions, including the overall VAS, pain recovery time, ulcer healing time, and natural and synthetic treatment-group analyses, were designated as secondary, exploratory syntheses during peer-review revision. The limited number of studies for individual products motivated these broader summaries but did not establish clinical exchangeability. Their interpretation was restricted to the particular mix of interventions included, without assuming a common therapeutic effect or applicability to individual products. Random-effects models allowed for between-study variation but did not resolve differences in mechanisms, formulations, treatment protocols, or outcome assessment. Heterogeneity and uncertainty were considered when interpreting each estimate. The natural and synthetic categories were descriptive groupings and were not treated as homogeneous therapeutic classes.

Heterogeneity was assessed using the I^2^ statistic, with values of 25%, 50%, and 75% interpreted as low, moderate, and high heterogeneity, respectively. In analyses with substantial heterogeneity, pooled estimates were interpreted cautiously, and prediction intervals were examined to reflect the range of effects expected in future studies. Subgroup analyses differentiated between natural and synthetic interventions. Potential publication bias was evaluated using funnel plots and Egger’s regression test; *p*-values < 0.05 indicated possible asymmetry.

The methodological quality of the included studies was assessed using the Cochrane Risk of Bias 2.0 tool, which evaluates domains such as sequence generation, allocation concealment, blinding, completeness of outcome data, and selective reporting. Although some studies were classified as high risk of bias, they were retained in the analysis unless they were found to contribute significantly to heterogeneity.

Forest plots and funnel plots were generated using the Meta-Analysis Online Tool (https://metaanalysisonline.com/; accessed on 13 August 2026), an open-access web-based platform that utilizes methods similar to those employed in Review Manager (RevMan) [28]. The tool enables random-effects modelling and provides a visual representation of effect sizes, heterogeneity, and publication bias.

To evaluate the robustness of the pooled estimates, a leave-one-out sensitivity analysis was conducted. In this approach, each study was sequentially excluded, and the meta-analysis was recalculated to assess whether any individual study had a disproportionate influence on the overall pooled effect size and heterogeneity. These analyses evaluated the numerical influence of individual studies on the exploratory estimates. Stability after study omission was not considered evidence that the interventions were clinically exchangeable.

A descriptive evidence profile was developed during manuscript revision to summarize the extent, risk of bias, and key findings of the evidence available for each intervention. Interventions were arranged alphabetically and grouped by the named agent or modality, with combination products presented separately from their individual components. Each study was counted once within its intervention category, regardless of the number of outcomes or assessment times reported. Stacked bars displayed the number of studies classified as having low risk of bias, some concerns, or high risk of bias, based on the overall judgments from the review’s risk-of-bias assessment.

Accompanying text summarized intervention-specific findings and their main limitations, distinguishing findings from individual trials from pooled estimates where reported. Discordant findings across studies, outcomes, or assessment times were described narratively rather than reduced to a single directional classification. Statistically non-significant differences were not interpreted as evidence of equivalence. Bar lengths represented study counts and were not weighted by sample size, effect magnitude, or statistical precision. Risk-of-bias judgments were presented separately from the concept of overall certainty of evidence; no formal certainty rating was assigned through this profile. The evidence profile was intended to describe the available research and its limitations, rather than to rank interventions or establish comparative efficacy or safety.

## 3. Results

Twenty-six randomized clinical trials were qualitatively evaluated. The follow-up duration across the included studies varied, with most studies assessing outcomes within a short period ranging from approximately 5 to 10 days, reflecting the typical healing course of recurrent aphthous ulcers, while one study reported a longer follow-up period of up to six months. Salvizan gel, Punica granatum and cannabinoid gel were compared with triamcinolone for efficacy in pain relief and overall healing. Salvizan gel, Punica granatum, and cannabinoid gel were shown to have better pain relief and healing than triamcinolone in the extracted data. Other effective remedies included laser therapies, such as diode and low-level lasers, and photo-modulation, which provide pain relief with no side effects; however, triamcinolone, low-level laser therapy (LLLT) provides better outcomes for pain reduction and ulcer healing [29,30]. Similarly, both pulsed and continuous carbon dioxide (CO_2_) lasers provided better pain relief than triamcinolone, although no significant difference in ulcer size reduction was observed among the treatment groups [31]. Further research with larger sample sizes and extended follow-up periods is necessary to assess the long-term recurrence rates and cost-effectiveness of laser therapy [32].

Amongst the natural topical agents, sage (Salvizan) gel reduced pain at 1.5 days compared with 2.5 days, and overall ulcer healing occurred at 3.3 days compared with 6 days in the triamcinolone group. This might be due to sage’s anti-inflammatory properties [33]. *Rhus coriaria* (Sumac) showed notable differences in pain reduction and ulcer size; however, these differences were not statistically significant compared to triamcinolone [34,35]. Punica granatum (pomegranate) gel and flower tablets have been shown to reduce pain and shorten healing time [26,36]. In one study, the tablets were directly placed on the lesion until they dissolved; therefore, this study was classified as a topical treatment [36]. Cannabidiol gel (CBD) reduced ulcer size and healing time, whereas triamcinolone accelerated healing [37]. Compared with triamcinolone, aloe vera showed a slight improvement at relieving pain and burning sensations, although the latter was more effective at promoting ulcer healing [38]. Curcumin has demonstrated efficacy comparable to that of triamcinolone in lowering pain and ulcer size [39,40,41]. The comparative evaluation of triamcinolone acetonide with honey-based treatments revealed superior pain reduction, ulcer healing, and patient satisfaction, while avoiding the side effects associated with corticosteroids [41,42,43,44,45]. A comparative evaluation of triamcinolone acetonide with other treatments for the management of RAS revealed that alternative treatments, including hyaluronic acid [46,47], microbiome therapy [48], triester glycerol oxide (TGO) [49], and polyherbal formulations [50], showed promising efficacy.

A comparison between Triadent (triamcinolone mucoadhesive paste) and Ora-Aid (a mucoadhesive patch) revealed that while Triadent patches were more effective at reducing lesion size over time, Ora-Aid provided superior relief from pain and burning sensations [51]. Compared with other topical treatments, triamcinolone and Amlexanox (5%) are significantly more efficacious in reducing the number and size of ulcers, as well as pain, erythema, and exudate levels [52,53] (Table 2).

However, to identify broader patterns, assess heterogeneity, and detect publication bias, a quantitative analysis is required.

### 3.1. Quantitative Results

The meta-analysis employed random-effects models to account for variability across studies.

For exploratory synthesis of VAS scores across interventions, 14 RCTs encompassing 836 patients with aphthous stomatitis, with 428 patients in the experimental group (Hiora SG gel, curcumin, thyme honey, *Punica granatum* flower, *Rhus coriaria*, honey, honey lemon, hyaluronic acid, amlexanox, triester glycerol oxide, and Ora-aid) and 408 in the control group (triamcinolone). Quantitative analysis was performed to compare the efficacy of topical triamcinolone with that of other topical treatments for the management of RAS. Efficacy was assessed by comparing pain relief using the visual analogue scale (VAS) and the pain recovery time.

**Figure 2:**
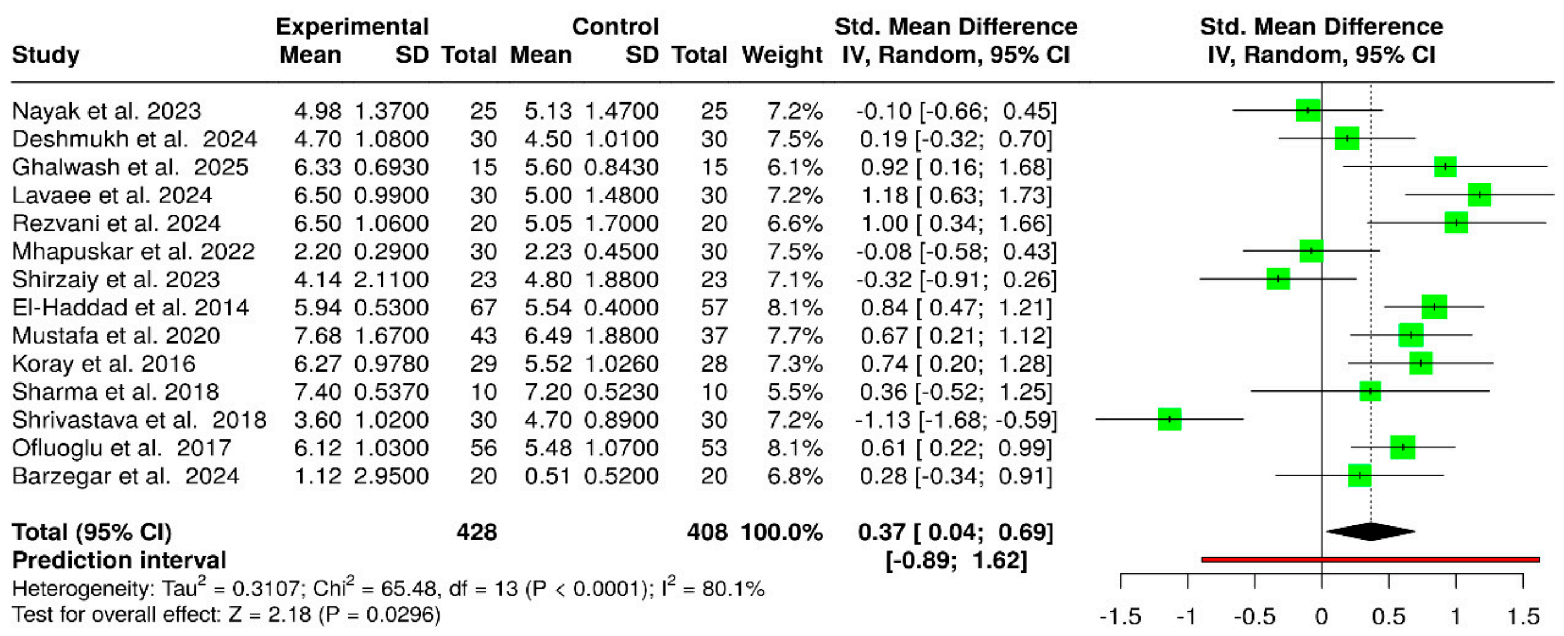
Exploratory random-effects meta-analysis of standardized mean differences in post-treatment visual analog scale (VAS) pain scores between the included interventions and topical triamcinolone. Refs. [32,34,36,42,43,44,45,46,47,49,50,51,52]. Green squares represent individual study effect estimates, with square size reflecting study weight; horizontal lines indicate 95% confidence intervals. The black diamond represents the pooled effect estimate and its 95% confidence interval, while the red horizontal line represents the 95% prediction interval.

This plot displays the mean difference in visual analog scale (VAS) scores between the experimental (Hiora SG gel, curcumin, thyme honey, *Punica granatum* flower, *Rhus coriaria*, honey, honey lemon, hyaluronic acid, amlexanox, triester glycerol oxide, and ora-aid) and control groups (triamcinolone), providing an estimate of the intervention’s effect on pain reduction compared with triamcinolone (Figure 2). The I^2^ value of 80% (95% CI 63–90%) indicates substantial heterogeneity, indicating that the variability in effect sizes among studies is substantial. Possible reasons for heterogeneity include differences in study populations (e.g., variations in age and pain severity), variability in intervention types (e.g., different formulations of experimental treatments), or differences in follow-up periods (e.g., some studies may have measured VAS reduction at various time points). The prediction interval ranged from −0.89 to 1.62, indicating that the true effect in future comparable studies may vary considerably and could include both benefit and no effect. A funnel plot was also used to review publication bias. The funnel plot did not indicate any asymmetry. Therefore, there was no publication bias.

**Figure 3:**
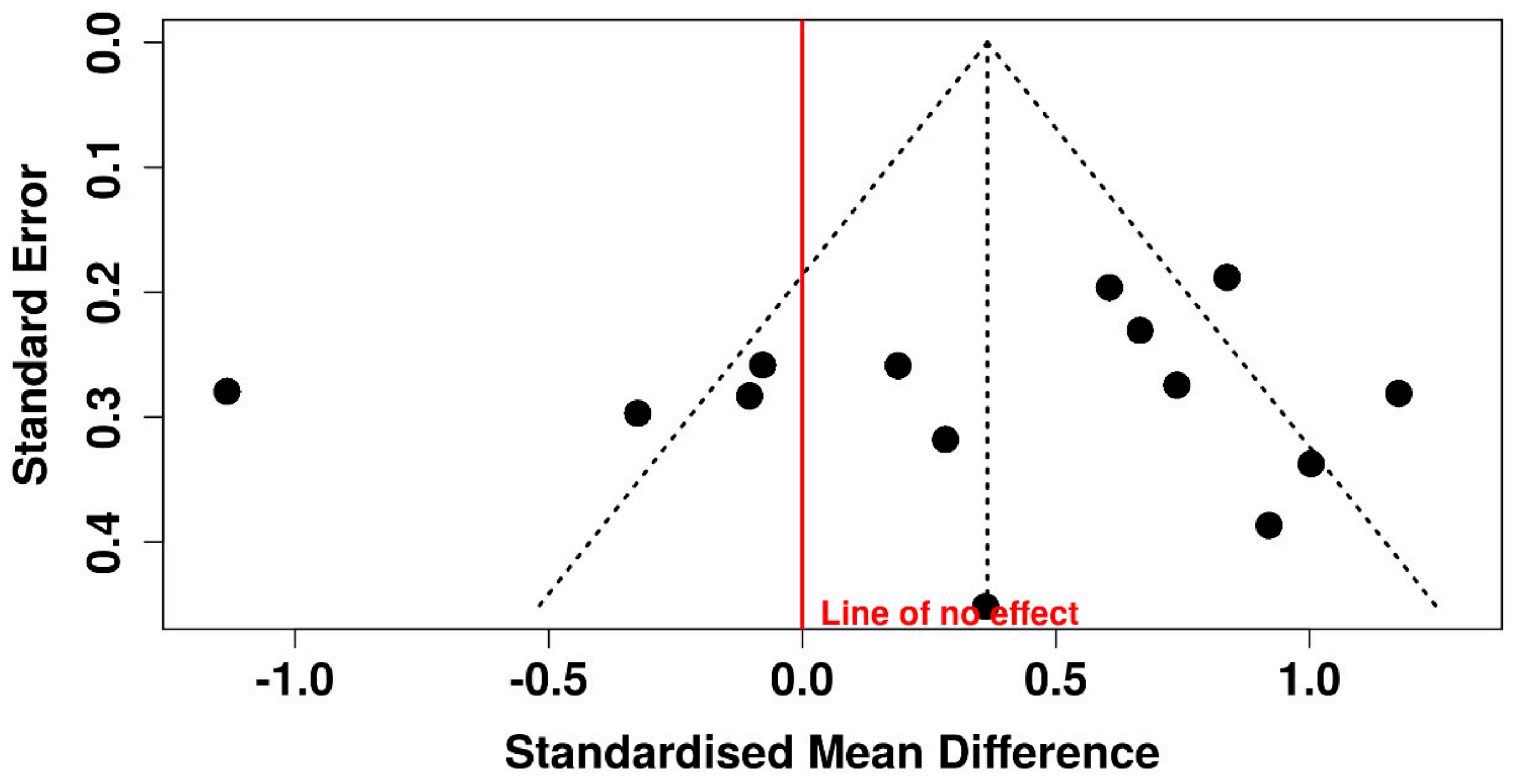
Funnel plot for standardized mean differences in VAS scores between the experimental and control groups. Black dots represent individual studies. The black dashed diagonal lines indicate the pseudo 95% confidence limits, while the black dashed vertical line represents the pooled effect estimate. The red vertical line indicates the line of no effect.

Additionally, Egger’s test did not indicate funnel plot asymmetry (*p* = 0.547). (Figure 3) Subgroup analyses were performed to differentiate the efficacy of natural versus synthetic agents.

**Figure 4:**
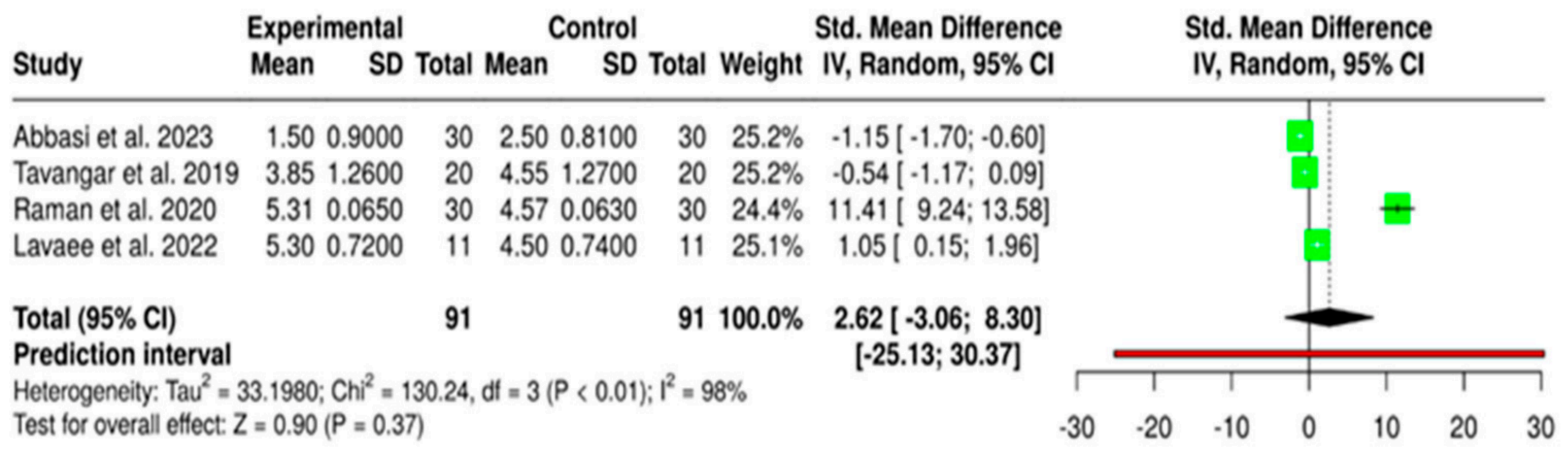
Exploratory random-effects forest plot of standardized mean differences in pain recovery time across different interventions compared with topical triamcinolone. Refs [26,33,36,39]. Green squares represent individual study effect estimates, with square size reflecting study weight; horizontal lines indicate 95% confidence intervals. The black diamond represents the pooled effect estimate and its 95% confidence interval, while the red horizontal line represents the 95% prediction interval.

An exploratory random-effects synthesis compared pain recovery time between the included interventions and topical triamcinolone. A forest plot included four studies reporting the mean pain recovery time of patients with RAS as an outcome, comparing the experimental (sage, Punica granatum, curcumin, and *Rhus coriaria*) and control groups (triamcinolone). A total of 182 patients were involved, with 91 in the experimental group and 91 in the control group. Based on the analysis performed using the random effects model with the inverse variance method to compare the standardized mean difference (SMD), there was no significant difference between the pain recovery times of the two groups, with a summarized standardized mean difference (SMD) of 2.62 and a *p*-value of 0.37 (Figure 4). The included interventions and definitions of pain recovery differed across studies, limiting the clinical meaning of the pooled estimate. The result should be interpreted as an exploratory summary of these studies rather than a common effect applicable to each treatment. Failure to detect a statistically significant difference does not establish equivalence between the interventions and triamcinolone.

Significant heterogeneity was detected, indicating inconsistent magnitudes and/or directions of effects. The I^2^ value suggests that 98% (95% CI: 92–99%) of the study variability arises from heterogeneity rather than random chance. Possible reasons for high heterogeneity include large variability in patient populations (e.g., different pain severity levels and underlying conditions), differences in pain recovery methods across studies, inconsistent intervention protocols (including dosage, duration, and type of experimental treatment), or outlier studies with disproportionately large effect sizes. The prediction interval ranged from −25.13 to 30.37, indicating very large uncertainty and suggesting that the effect in future studies may vary widely in magnitude and direction.

**Figure 5:**
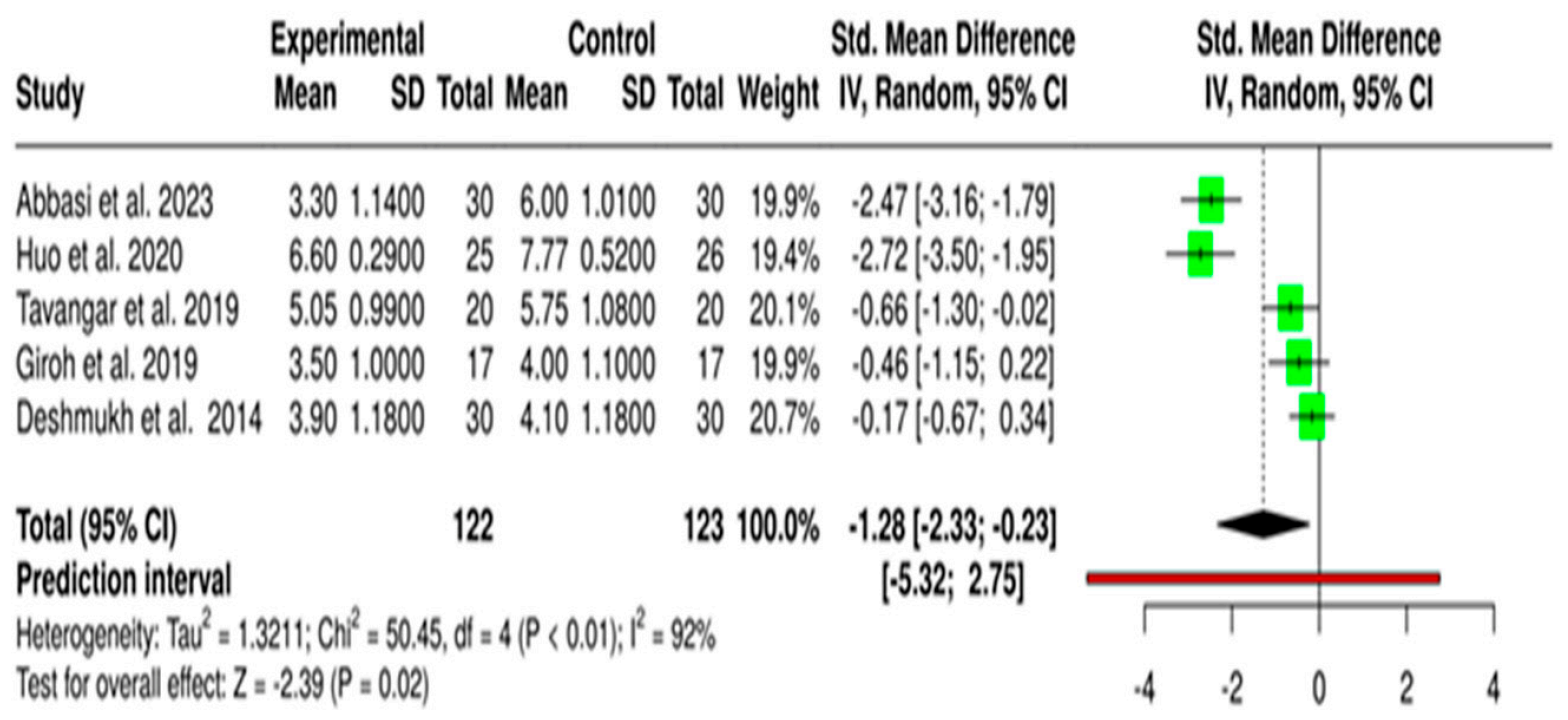
Exploratory random-effects forest plot of standardized mean differences in ulcer healing time across different interventions compared with topical triamcinolone. Refs. [26,29,33,38,41]. Green squares represent individual study effect estimates, with square size reflecting study weight; horizontal lines indicate 95% confidence intervals. The black diamond represents the pooled effect estimate and its 95% confidence interval. The red horizontal line represents the 95% prediction interval. The solid vertical line indicates the line of no effect, and the dotted vertical line indicates the pooled effect estimate.

An exploratory random-effects synthesis compared ulcer healing time between the included interventions and topical triamcinolone. Five RCTs encompassing a total of 245 patients with RAS were analyzed, with 122 patients in the experimental group (treated with sage, diode laser, Punica granatum, aloe vera, and curcumin) and 123 in the control group (treated with triamcinolone), compared with the mean difference in ulcer healing time among patients. The exploratory average −1.28 (−2.33, −0.23) favoured the included interventions, although this finding cannot be generalized to individual products. (Figure 5).

The high heterogeneity (I^2^ = 92%, 95% CI 78–97%) suggests that the pooled estimate should be interpreted with caution. Possible reasons for high heterogeneity include variability in sample sizes and patient demographics across studies, differences in intervention methods (e.g., dosage, frequency, administration method), and measurement inconsistencies in recorded outcomes (e.g., variations in how pain or healing was assessed). The prediction interval ranged from −5.32 to 2.75, suggesting that the magnitude and direction of effect may vary across future studies.

**Figure 6:**
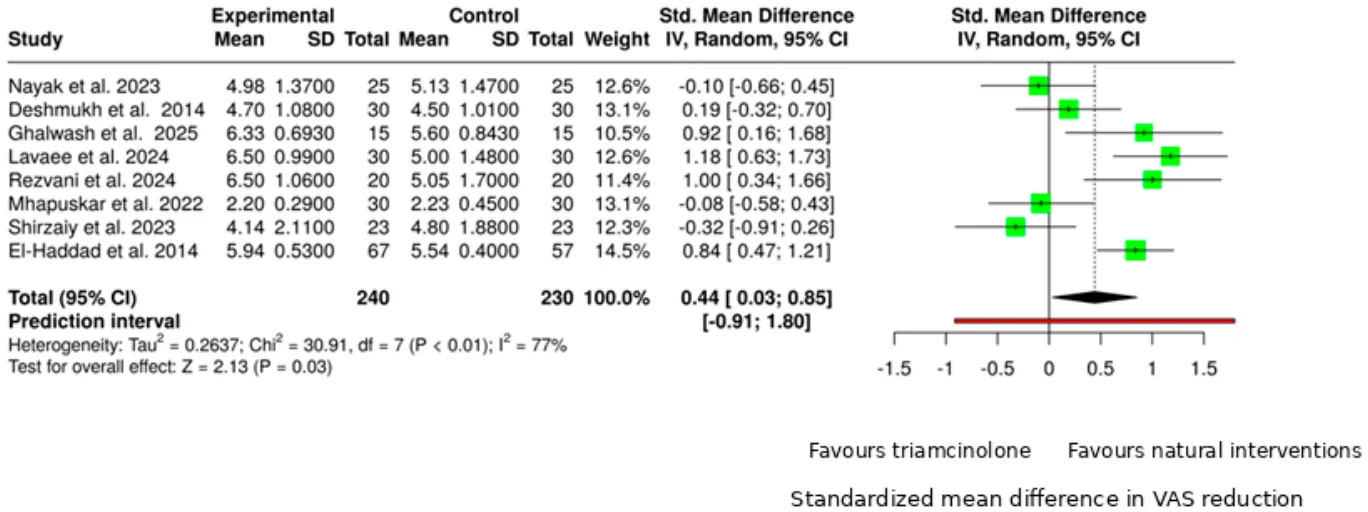
Exploratory random-effects meta-analysis of standardized mean differences in VAS pain reduction from baseline between natural interventions and topical triamcinolone. Pain reduction was calculated as baseline minus follow-up VAS. Positive SMDs favour the natural intervention, and negative SMDs favour triamcinolone. Refs. [32,34,35,41,42,43,45,50]. Green squares represent individual study effect estimates, with square size reflecting study weight; horizontal lines indicate 95% confidence intervals. The black diamond represents the pooled effect estimate and its 95% confidence interval. The red horizontal line represents the 95% prediction interval. The solid vertical line indicates the line of no effect, and the dotted vertical line indicates the pooled effect estimate.

An exploratory synthesis examined VAS pain scores for interventions categorized as natural versus topical triamcinolone. This category comprised distinct products, including honey preparations, curcumin, Punica granatum, *Rhus coriaria*, HiOra SG, and honey–lemon. Overall, eight studies were analyzed, involving 470 subjects with aphthous stomatitis, 240 subjects in the experimental cohort, and 230 subjects in the control cohort. According to the analysis performed using the random effects model, a significant difference was found between the two cohorts; The exploratory pooled SMD for VAS reduction from baseline was 0.44, with the positive estimate indicating greater mean pain reduction in the natural-intervention groups. This standardized estimate does not itself establish a clinically important difference. Significant heterogeneity was detected, indicating inconsistent magnitudes and/or directions of effects. The I^2^ value suggests that 77% (95% CI 48–90%) of the study variability arises from heterogeneity rather than random chance (Figure 6). The prediction interval ranged from −0.91 to 1.80, indicating that the true effect in future studies may vary and could include both benefit and no effect.

**Figure 7:**
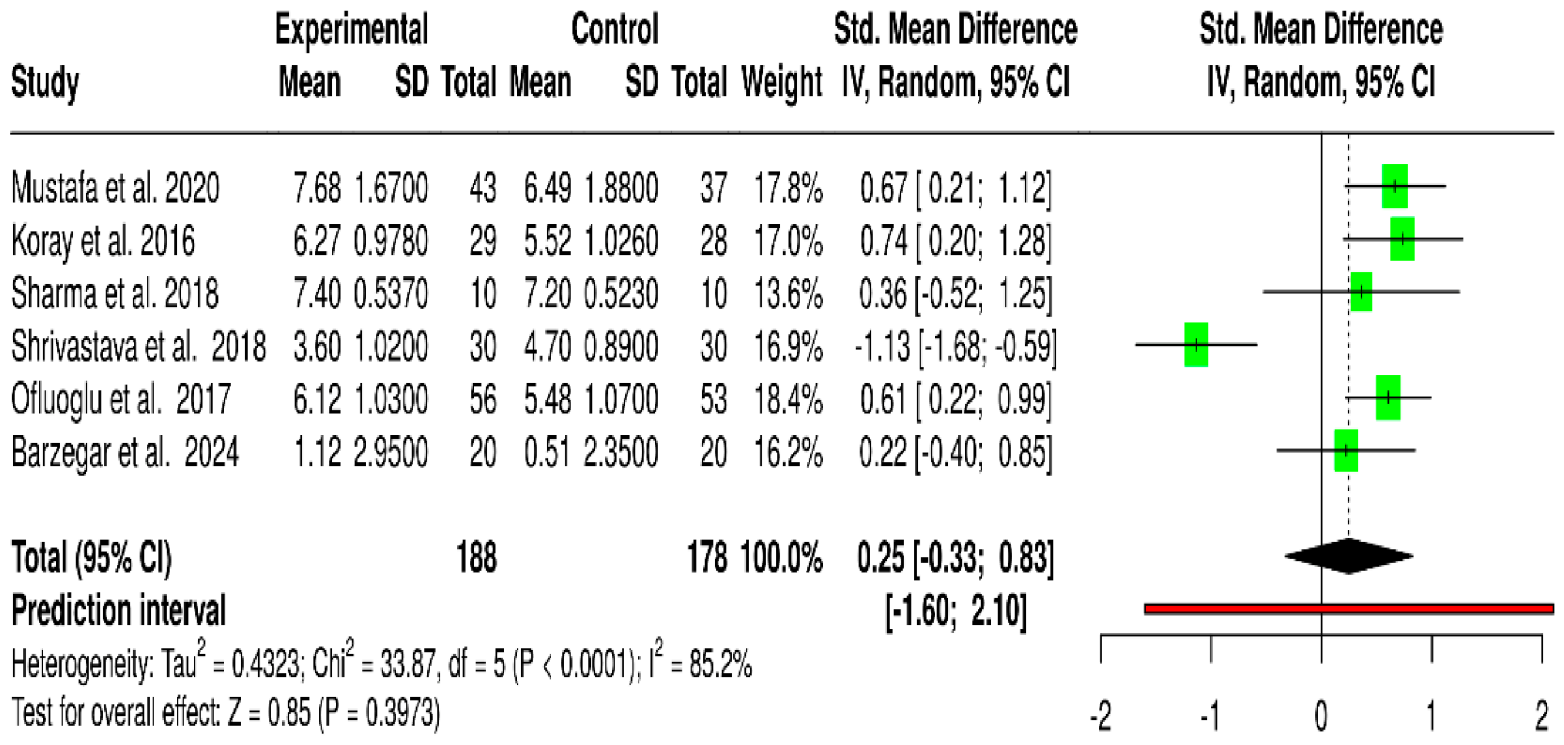
Exploratory random-effects forest plot of standardized mean differences in VAS pain scores across distinct synthetic interventions compared with topical triamcinolone. Refs. [46,47,52,53,49,51]. Green squares represent individual study effect estimates, with square size reflecting study weight; horizontal lines indicate 95% confidence intervals. The black diamond represents the pooled effect estimate and its 95% confidence interval. The red horizontal line represents the 95% prediction interval. The solid vertical line indicates the line of no effect, and the dotted vertical line indicates the pooled effect estimate.

An exploratory synthesis of the mean difference in visual analog scale (VAS) scores between the synthetic experimental groups (hyaluronic acid, amlexanox, triester glycerol oxide, or Ora-aid) and control groups (triamcinolone) was performed, providing an estimate of the effect of the synthetic intervention on pain reduction compared with that of triamcinolone. A total of 6 studies were included, encompassing 366 patients with aphthous stomatitis:188 in the experimental group and 178 in the control group. The standardized mean difference from the random effects forest plot was 0.25 (−0.33, 0.83), suggesting that the synthetic intervention reduced pain intensity in patients more than did triamcinolone, but the difference was not significant. (Figure 7). The I^2^ value indicates that 85% (95% CI 67–94%) of the variability among studies arises from heterogeneity rather than random chance. The prediction interval ranged from −1.60 to 2.10, suggesting that the true effect in future studies may vary substantially and could include both benefit and no effect.

The above plot indicates that some studies show triamcinolone to be more effective, while others suggest that synthetic topical agents are more effective. The pooled effect is not statistically significant due to high heterogenicity. A few studies were excluded from the quantitative analysis because their outcomes did not meet the criteria for the parameters in the meta-analysis [30,31,32,37,40,48].

A funnel plot was not generated because there were fewer studies on pain recovery and ulcer healing time.

A subgroup meta-analysis was conducted to compare triamcinolone acetonide with honey-based natural therapies (including honey, thyme honey, and honey–lemon preparations) for pain reduction. Four studies were included in this analysis, comprising a total of 260 participants (135 in the triamcinolone group and 125 in the honey-based therapy group) [42,43,44,45].

**Figure 8:**
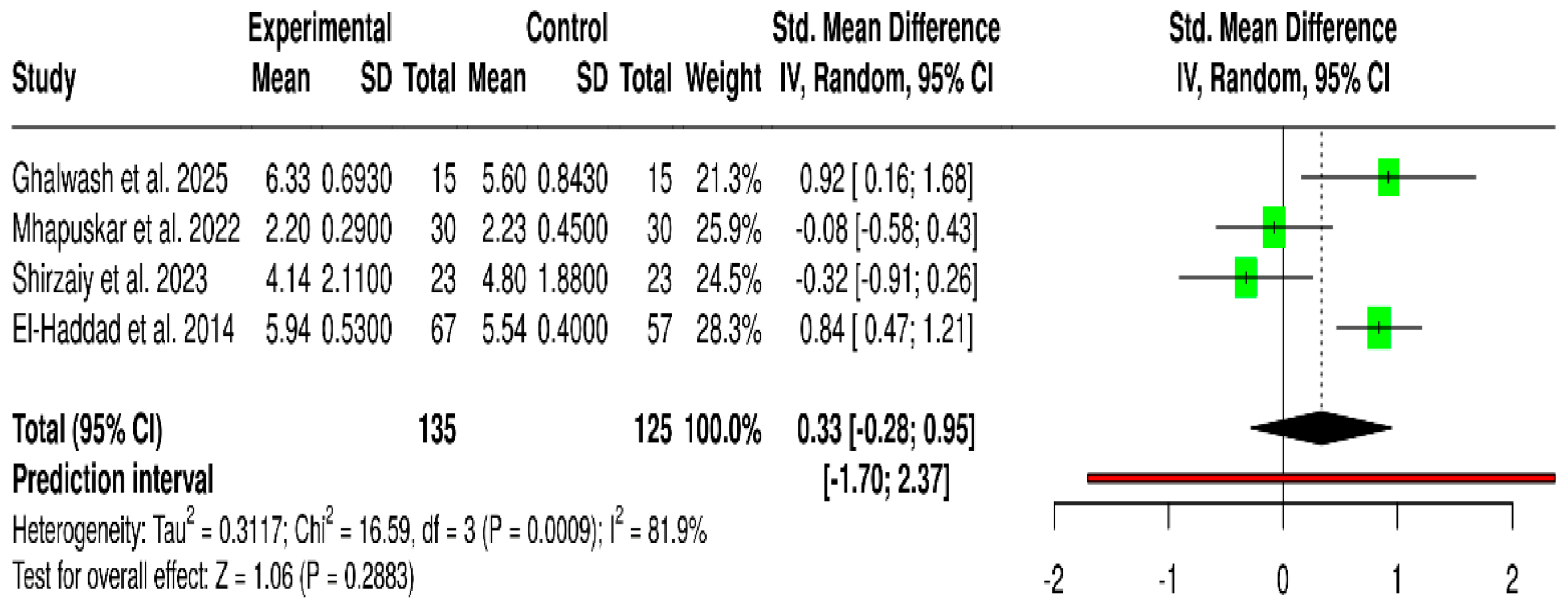
The pooled standardized mean difference using a random-effects model in VAS in triamcinolone and honey-based. Refs. [42,43,44,45].

As shown in Figure 8, the pooled standardized mean difference using a random-effects model showed no statistically significant difference between triamcinolone and honey-based treatments (SMD = 0.33, 95% CI: −0.28 to 0.95; *p* = 0.29). Substantial heterogeneity was observed among the included studies (I^2^ = 81.9%, *p* = 0.0009), with a prediction interval ranging from −1.70 to 2.37. Despite the heterogeneity, several individual studies demonstrated comparable or favorable pain reduction with honey-based interventions, suggesting that honey-based natural therapies may represent a promising alternative to corticosteroid therapy for symptom relief.

A subgroup meta-analysis was conducted to compare triamcinolone acetonide with amlexanox for pain reduction. Two studies were included in this analysis, comprising a total of 80 participants (40 in the triamcinolone group and 40 in the amlexanox group) [52,53].

**Figure 9:**
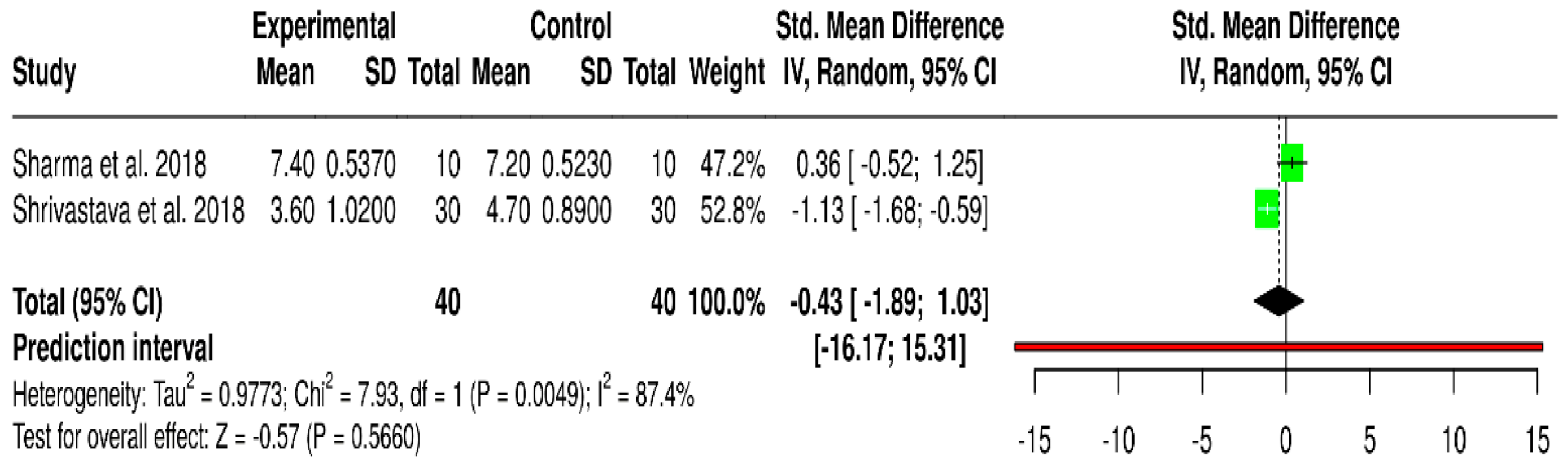
The pooled standardized mean difference using a random-effects model between triamcinolone and amlexanox. Refs. [52,53].

As shown in Figure 9, the pooled standardized mean difference using a random-effects model demonstrated no statistically significant difference between triamcinolone and amlexanox (SMD = −0.43, 95% CI: −1.89 to 1.03; *p* = 0.57). Substantial heterogeneity was observed between the studies (I^2^ = 87.4%, *p* = 0.0049), and the prediction interval ranged widely from −16.17 to 15.31, indicating considerable variability in the possible true effects. Given the limited number of studies and the high heterogeneity, these findings should be interpreted with caution. Nevertheless, the pooled estimate suggests that amlexanox may provide a level of pain reduction comparable to triamcinolone, although further well-designed studies are needed to confirm these findings.

A subgroup meta-analysis was conducted to compare triamcinolone acetonide with hyaluronic acid for pain reduction. Two studies were included in this analysis, comprising a total of 137 participants [46,47].

**Figure 10:**
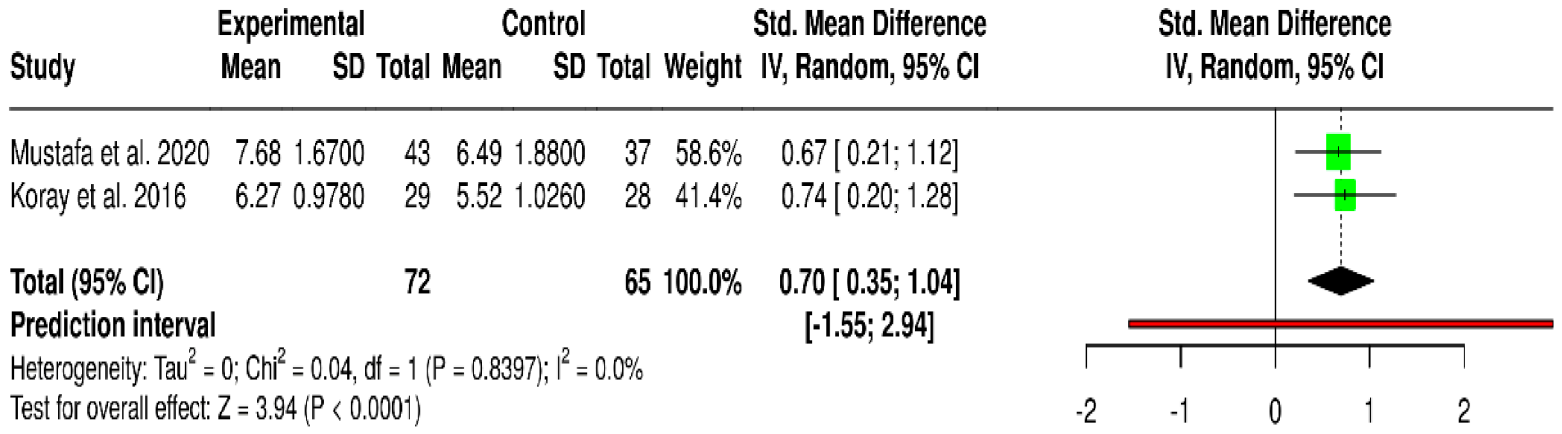
Pooled standardized mean difference using a random-effects model difference between triamcinolone acetonide with hyaluronic acid for pain reduction. Refs. [46,47].

As shown in Figure 10, the pooled standardized mean difference using a random-effects model showed a statistically significant difference between the two treatments (SMD = 0.70, 95% CI: 0.35 to 1.04; *p* < 0.0001). No heterogeneity was observed among the included studies (I^2^ = 0%, *p* = 0.84), indicating consistent findings across studies. The prediction interval ranged from −1.55 to 2.94. Given that only two studies were included, the findings should be interpreted with caution.

The certainty of the evidence for the assessed outcomes was evaluated by examining study quality, consistency of findings, precision of the effect estimates, and the potential for publication bias across the included randomized controlled trials. The primary pooled analysis showed a small but statistically significant overall effect (SMD = 0.37; 95% CI 0.04–0.69; *p* = 0.0296). However, the presence of substantial heterogeneity (I^2^ = 80.1%) reduced confidence in the estimate, suggesting a moderate level of certainty.

#### 3.1.1. Sensitivity Analysis

To assess the influence of individual studies on heterogeneity, we performed leave-one-out analyses targeting outlier trials identified by the authors.

#### 3.1.2. Pain Visual Analog Scale (Figure 2)

After removing the Shrivastava et al. study [53], heterogeneity dropped substantially from I^2^ = 84% to 64%, while the overall pooled effect size remained significant and consistent in direction (SMD = 0.48, 95% CI: 0.22–0.73, *p* = 0.0002). This indicates that although the Shrivastava trial contributed to heterogeneity, it did not alter the overall conclusion.

#### 3.1.3. Pain Recovery Time

Exclusion of the Raman et al. study [39] reduced heterogeneity from I^2^ = 98% to 92%. The effect estimate remained non-significant (SMD = 2.74, 95% CI: −0.20 to 5.69, *p* = 0.067), suggesting that variability persisted after study removal; however, the effect’s direction remained unchanged.

#### 3.1.4. Synthetic Topical Application Subgroup Figure 7

Excluding Shrivastava et al. [53] reduced heterogeneity dramatically from I^2^ = 85% to 0%, with a consistent and significant pooled effect (SMD = 0.58, 95% CI: 0.35–0.81, *p* < 0.0001).

As shown in the forest plots, some outcomes demonstrated substantial heterogeneity, with I^2^ values exceeding 75%. This variation likely reflects differences among the included studies, such as variations in ulcer types, treatment formulations, dosage regimens, outcome measures, and follow-up durations.

The sensitivity analyses showed that the estimated heterogeneity varied following study omission, with the extent of the change differing between analyses. The remaining studies continued to include different interventions and treatment protocols. The pooled estimates were therefore interpreted as exploratory, including analyses in which the estimated heterogeneity decreased substantially.

### 3.2. Risk of Bias

**Figure 11:**
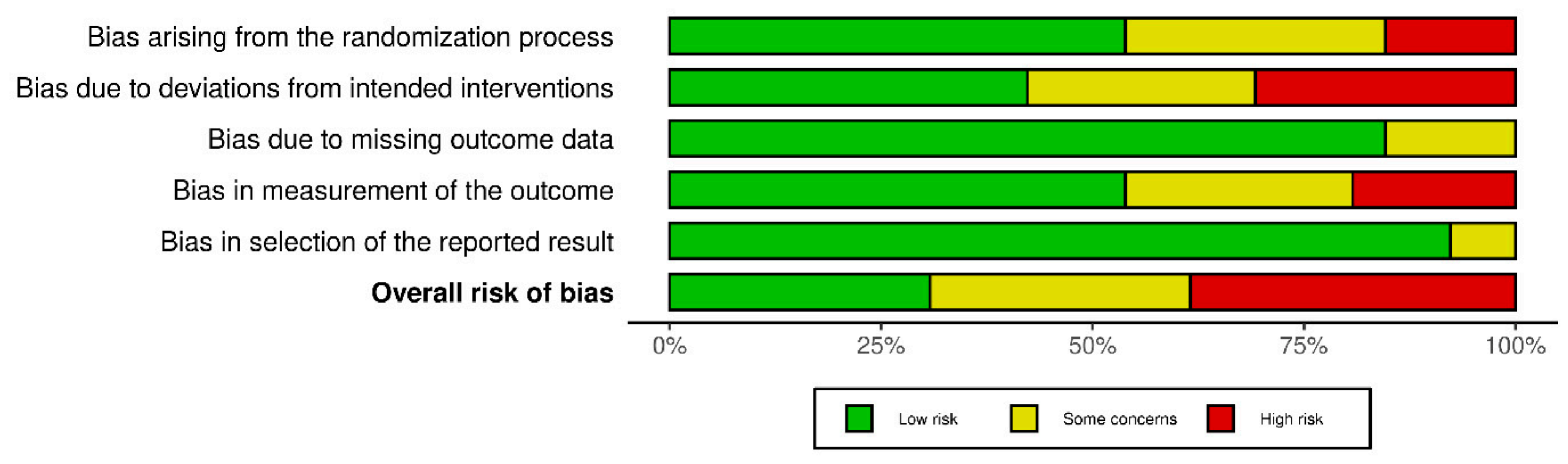
Summary of RoB 2.0 domain-level risk of bias across included randomized controlled trials.

**Figure 12:**
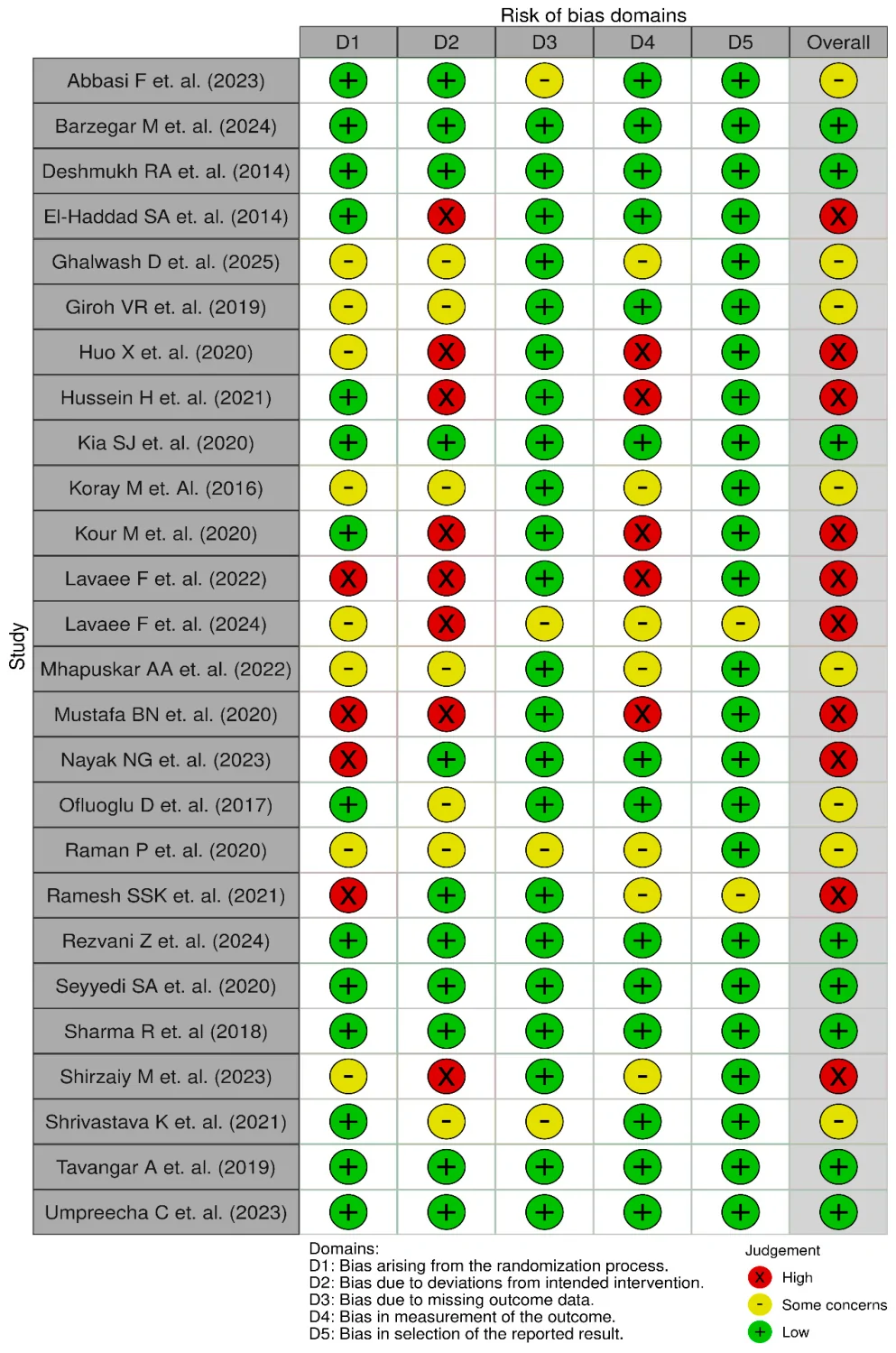
RoB 2.0 “traffic light” plot showing risk of bias judgements by study across domains D1–D5 and overall [33,51,41,45,42,38,29,30,40,47,31,34,36,43,46,50,49,39,48,35,32,52,44,53,26,37].

The risk of bias assessment was performed to evaluate the transparency of the evidence synthesis results and findings. Each of the selected studies was included for risk of bias assessment (Figure 11 and Figure 12).

**Figure 13:**
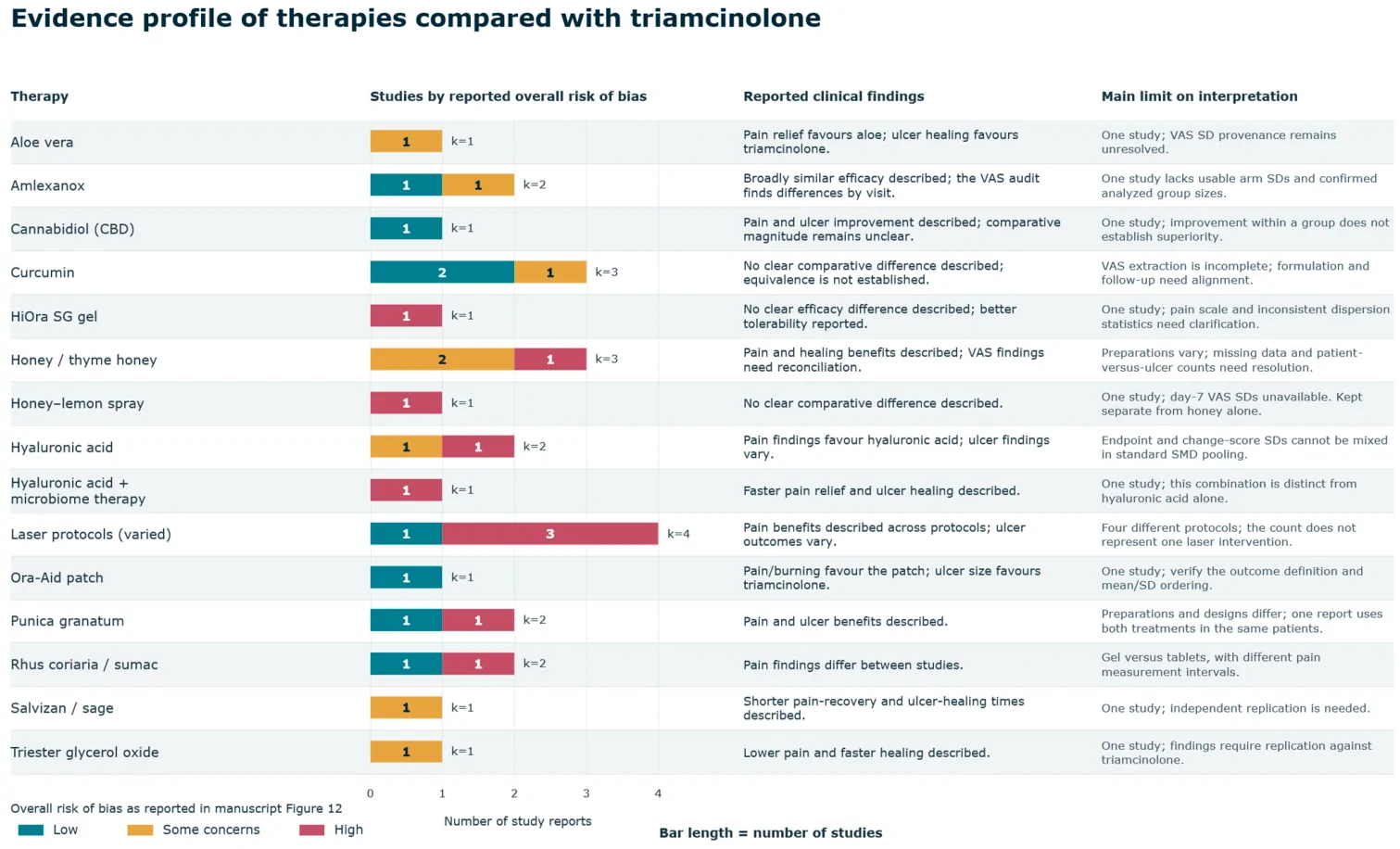
Descriptive evidence profile of interventions compared with topical triamcinolone for recurrent aphthous stomatitis. Stacked bars show the number of included study reports within each intervention category, subdivided by the overall risk-of-bias judgments reported in Figure 12. Numbers within segments indicate study counts, and k denotes the total number of reports in each category. Accompanying text summarizes reported clinical findings and limitations. Categories containing different preparations or protocols are grouped for descriptive purposes only. Bar length and colour do not indicate treatment effectiveness, sample size, precision, or certainty of evidence. The figure does not provide a treatment ranking or a formal GRADE assessment.

The risk of bias of the included randomized controlled trials was evaluated using the RoB 2.0 tool to assess the reliability and transparency of the synthesized evidence (Figure 11 and Figure 12). Overall, most studies demonstrated a low risk of bias across several domains, particularly for missing outcome data (D3) and selection of the reported results (D5), where the majority of trials were judged as low risk. However, some concerns and high-risk judgments were observed in domains related to deviations from intended interventions (D2) and measurement of outcomes (D4), indicating potential methodological limitations in a subset of studies. The overall assessment showed that while many studies were considered low risk, several presented some concerns or high risk of bias, highlighting variability in methodological rigor across the included trials. Figure 13 summarizes the number of study reports available for each intervention category, their reported overall risk of bias, and the principal clinical findings and limitations.

The evidence was unevenly distributed across interventions, with several products evaluated in only one study. Reported risk of bias also varied between studies. Figure 13 makes these differences visible while presenting clinical findings descriptively. Larger bars indicate more study reports and should not be interpreted as greater treatment benefit or stronger certainty of evidence. The figure should be considered alongside the individual-study results and any appropriate treatment-specific meta-analyses.

## 4. Discussion

Triamcinolone is an anti-inflammatory agent that inhibits the release of pro-inflammatory mediators; hence, it is a common topical treatment for RAS [7,10]. However, alternative effective natural and synthetic treatments have gained attention because of the potential side effects of corticosteroids. Triamcinolone has been reported to have associated side effects such as dry mouth, candidiasis, nausea, immune suppression, fatigue, fluid retention, and osteoporosis, impeding hormonal balance and digestive troubles [11,42,43,54]. Due to the numerous side effects, more alternative options have gained patient acceptance [55]. Several natural topical treatments, such as honey [23,43,45], sage (*Salvia officinalis*) [33], chamomilla [20], pomegranate (Punica granatum) extracts [10,25,26,36,56], curcumin [39,40,41], and aloe vera [19,38] have demonstrated comparable efficacy to corticosteroids, with no side effects. In addition, synthetic alternatives such as low-level laser therapy (LLLT) [12,29,30], hyaluronic acid [18,46,47], microbiome-based treatments [48] and mucoadhesive patches [51] have effectively reduced pain and promoted healing. Substantial heterogeneity was observed across the pooled analyses, reflecting differences in treatment mechanisms, formulations, dosage regimens, and follow-up periods. Although the interventions addressed the same clinical condition and were compared with topical triamcinolone, their effects on pain and healing may differ. The limited number of studies available for individual products motivated a broader synthesis to provide an overview of the available evidence. Accordingly, the overall and treatment-category estimates are interpreted as exploratory. These summaries should be considered alongside the findings for each intervention, particularly where studies differ in outcome assessment or risk of bias. Further trials of individual products using consistent treatment protocols and assessment times are needed to establish their comparative benefits.

When evaluating patient satisfaction, treatments such as *Rhus coriaria*, honey, curcumin, aloe vera, and *Punica granatum* resulted in greater satisfaction than corticosteroids, with no adverse reactions [35,36,37,40,41,44,45,46]. This finding is similar to those of many other studies [10,23,56,57,58]. Compared to triamcinolone, synthetic alternatives, such as laser therapy and hyaluronic acid, were reported to be effective in overall healing, including reducing the recurrence of aphthous stomatitis for a longer period [29,30,31,32,46,47,48]. This finding is consistent with the conclusions of other authors [10,55,59,60]. The ability of cannabidiol (CBD) gel to reduce ulcer size and healing time, albeit with slower healing than triamcinolone, is a novel finding [37]. The better pain relief and ulcer healing reported with *Punica granatum*, cannabinoid gel, and honey in some studies may be related to differences in their biological effects. These treatments contain naturally occurring compounds that have been associated with anti-inflammatory, antioxidant, analgesic, and tissue-healing effects, which may help reduce inflammation and support mucosal repair. Triamcinolone, although effective in controlling inflammation, may affect some cellular processes involved in tissue repair when exposure is prolonged or excessive, including fibroblast activity and collagen production. These differences may partly account for the findings reported in individual studies [61]. The VAS analysis in Figure 2 provides an exploratory summary of differences in pain reduction between the included interventions and triamcinolone. Its clinical interpretation requires consideration of the individual treatments, baseline pain severity, and timing of assessment. The pooled SMD alone does not establish whether the difference would be meaningful to patients, and the average result may not represent the benefit associated with a particular intervention. Some studies [36,51] have reported substantial benefits of alternatives. Conversely, few studies have reported minimal or negative effect sizes, suggesting potential variability in response depending on the agent used and the patient population [43,44,53].

Among synthetic alternatives, low-level laser therapy (LLLT) and other laser-based modalities, such as diode and CO_2_ lasers, provide rapid pain relief with minimal side effects [12,29,30,31,32]. Nonetheless, given the noninvasive and reproducible nature of laser treatments [12], they offer valuable options for patients seeking steroid-free alternatives, especially in cases of recurrent or refractory ulcers [62,63]. Similarly, another study found that combining laser therapy with hyaluronic acid resulted in faster healing and greater pain relief than triamcinolone [64]. HA, either alone or combined with microbiome-based treatments, has demonstrated superior pain control and mucosal healing outcomes, likely due to its bioadhesive and regenerative properties [18]. This finding is consistent with observations from a systematic review [60]. Interestingly, topical application of a novel formulation of triester glycerol oxide (TGO) outperformed triamcinolone in pain reduction and ulcer healing, with an easily applicable, accessible procedure and no side effects, suggesting a potential future role in mucosal protection and repair [49]. The sensitivity analyses showed that some pooled findings were influenced by individual studies. Reductions in heterogeneity following study omission highlight the importance of examining the characteristics and methodological limitations of influential trials. However, a lower I^2^ does not eliminate differences in the treatments being evaluated. These findings therefore support cautious interpretation of the exploratory pooled estimates, with greater emphasis on the evidence for each intervention. The study suggests that alternative topical therapies may achieve comparable or improved pain relief and healing outcomes relative to triamcinolone. Nevertheless, confidence is limited by substantial heterogeneity and small sample sizes. Table 3 lists all natural and alternative topical medications compared with triamcinolone in this study.

A summary of the diagnostic pathway, along with management and treatment pathways, is added in Table 4.

### 4.1. Limitations

This review evaluates a broad range of interventions compared with topical triamcinolone. The interventions differ in mechanism, formulation, administration, and treatment duration, while studies differ in design and outcome assessment. These differences limit clinical exchangeability and the meaning of an overall pooled effect. Random-effects modelling allows statistical variation but does not overcome this conceptual limitation. All retained analyses combining different interventions are therefore exploratory and cannot support treatment-class recommendations.

The limited number of studies and incomplete or incompatible outcome data constrain treatment-specific synthesis. Broader pooling does not resolve this lack of direct evidence for individual products. Prediction intervals, where estimable, describe additional uncertainty but do not make distinct interventions interchangeable. Findings remain dependent on the treatments and study populations represented in the available evidence.

Variability in methodological quality and reporting across the included studies also presented challenges during data synthesis. In several trials, details regarding randomization methods, allocation concealment, or blinding procedures were limited or unclear, which may introduce potential bias and affect confidence in the reported findings.

Overall, while the review provides valuable evidence regarding the comparative effectiveness of topical triamcinolone and other topical treatments, these limitations indicate that the results should be interpreted cautiously. Future studies with larger sample sizes, standardized outcome measures, longer follow-up periods, and more rigorous methodological reporting are needed to strengthen the evidence base.

### 4.2. Clinical Implications

The findings highlight several interventions that warrant consideration in the assessment of treatment options for recurrent aphthous stomatitis. Their clinical relevance depends on the evidence available for the particular product, formulation, and outcome being evaluated. Treatment decisions should consider ulcer severity, previous treatment response, tolerability, patient preferences, and practical factors such as availability and cost. Where an alternative to triamcinolone is being considered, patients should be informed about the extent and limitations of the supporting evidence. The exploratory pooled findings provide an overview of the available research but are insufficient to recommend natural or synthetic interventions collectively as first-line treatments. Further evidence is needed to establish comparative safety and effectiveness with repeated or prolonged use.

## 5. Conclusions

This systematic review identified a range of interventions evaluated against topical triamcinolone for recurrent aphthous stomatitis. Some individual studies reported favourable outcomes for pain relief or ulcer healing, although the findings varied by intervention and outcome. The limited number of studies for individual products, differences in treatment protocols, and methodological limitations restrict the strength of treatment-specific conclusions.

The pooled analyses across different interventions provide exploratory summaries and should be interpreted alongside the individual-study findings. They do not establish a consistent benefit or equivalent efficacy across all alternatives to triamcinolone. Further adequately powered trials of clearly defined interventions, using standardized outcome measures and assessment times, are needed. Longer follow-up and systematic reporting of adverse events, recurrence, and patient-reported outcomes would strengthen the evidence available for clinical decision-making.

## Figures and Tables

**Table 1:** Search strategy for systematic review.

1	Aphthous Stomatitis OR Stomatitis, Aphthous OR aphthous ulcer*.tw. OR canker sore*.tw. OR recurrent aphthous stomatitis.tw. OR mouth ulcer*.tw. OR oral ulcer*.tw.
2	Triamcinolone OR triamcinolone acetonide.tw. OR triamcinolone paste.tw. OR Kenalog.tw. OR Corticosteroid OR corticosteroid*.tw.
3	(Aloe vera OR chamomile OR licorice OR *Glycyrrhiza glabra* OR honey OR bee propolis OR cannabidiol OR CBD OR hyaluronic acid OR salvia OR sage OR curcumin OR turmeric OR *Rhus coriaria* OR sumac OR *Punica granatum* OR pomegranate OR amlexanox OR benzocaine OR lidocaine OR doxycycline OR probiotics OR chlorhexidine OR gel OR triester glycerol oxide OR polyherbal).tw.
4	Laser Therapy OR Low-Level Light Therapy OR Laser OR diode laser.tw. OR low-level laser.tw. OR carbon dioxide laser.tw. OR CO_2_ laser.tw. OR photobiomodulation.tw.
5	randomized controlled trial OR controlled clinical trial OR randomized.tw. OR randomized.tw. OR RCT.tw. OR placebo.tw. OR clinical trial.tw.
6	1 AND (2 OR 3 OR 4) AND 5
7	Limit 6 to (English language and humans and year = “March 2014–March 2025”)

**Table 2:** Summary of studies included in the qualitative analysis.

Authors	Sample Size	Follow-Up Duration	Efficacy of Triamcinolone (TA)	Efficacy of Compared Alternative Treatment
Ghalwash et al. (2025) [42]	30 patients (15 per group)	7 days	Reduced pain and ulcer size, but slower healing than thyme honey	Thyme honey showed greater ulcer size reduction on days 5 and 7, and greater pain relief on days 3, 5, and 7
Rezvani et al. (2025) [35]	60	7 days	Triadent took the longest time to heal ulcers.	The sumac group exhibited the shortest healing time, significantly reduced lesion size, and had lower Visual Analog Scale scores.
Barzegar et al. (2024) [51]	40 patients	7 days	Triadent was more effective in reducing ulcer size over time	Ora-Aid was better for pain and burning sensation relief
Lavaee et al. (2024) [36]	60	6 days	Triadent was less effective.	*Punica granatum* was significantly better than TA for all mean values of VAS & size of the oral lesions on all days except on day 1 for ulcer size.
Abbasi et al. (2023) [33]	60 (30 each)	8 days	The mean duration of pain recovery is 2.5 days for TA The duration of ulcer healing is 6 days for TA	The mean duration of pain recovery is 1.5 days, and the mean duration of ulcer healing is 3.3 days for Salvizan.
Nayak et al. (2023) [50]	50	10 days	Effective but had some side effects	HiOra SG gel was equally effective and better tolerated
Shirzaiy et al. (2023) [44]	46 patients (23 per group)	7 days	Reduced pain, burning sensation, necrosis, and erythematous area	Honey-lemon spray had similar efficacy to triamcinolone and was well-tolerated
Umpreecha et al. (2023) [37]	69	7 days	Both groups significantly reduced ulcer size TA as the most preferred intervention, followed by CBD.	CBD reduced ulcer size and accelerated ulcer healing without side effects. CBD exerted anti-inflammatory effects in the early stage and an analgesic effect in the late RAS stage. Thus, CBD might be more appropriate for RAS patients who decline to take topical steroids.
Lavaee et al. (2022) [34]	22 (11 each)	6 days	There were no significant differences	TA reduced pain more effectively, but ulcer healing was slightly better for *Rhus coriaria*
Mhapuskar et al. (2022) [43]	60 patients (30 per group)	7 days or until complete healing	Effective in reducing pain and ulcer size but associated with mild side effects	Honey effectively reduced ulcer size and pain with better patient compliance and no side effects.
Huo et al. (2021) [29]	56	7 days for laser group & until healing of RAS in medication group	The effect of pain relief and ulcer healing was slower	Laser therapy showed significantly faster pain reduction and ulcer healing
Hussein et al. (2021) [30]	28	7 days	Effective but slower than laser treatment	Low-level laser therapy (LLLT) provided faster pain relief and better healing
Ramesh & Sadaksharam (2021) [48]	45	7 days	Effective but slower healing than combination therapy	HA + microbiome therapy showed faster healing and better pain relief
Kia et al. (2020) [40]	58	10 days	No statistically significant differences	Curcumin is as effective as 0.1% TA and can be used in patients who are contraindicated to corticosteroids.
Kour & Dwivedi (2020) [31]	45	6 days	Effective but slower healing than lasers	The noncontact diode laser was the most effective in pain relief and ulcer healing
Mustafa & Alzubaidee (2020) [46]	80	6 days	Effective but less rapid pain relief than HA	Hyaluronic acid (HA) provided greater pain relief and faster healing
Raman et al. (2020) [39]	60	7 days & 6 months	TA reduced the mean ulcer size & pain significantly.	Curcumin is a better choice in children, pregnant women, lactating mothers, and immunocompromised individuals due to their safety.
Seyyedi et al. (2020) [32]	15	7 days	Effective in ulcer size reduction	CO_2_ laser provided superior pain relief, but no significant difference in ulcer size reduction
Giroh et al. (2019) [38]	34	7 days	TA is more effective than aloe vera in ulcer healing	Aloe vera gel showed a better response in terms of pain relief and reduction in burning sensation.
Tavangar et al. (2019) [26]	60	5 days	Less effective compared to alternative	Ulcer healing time and pain relief in the *Punica granatum* group were significantly lower than those of triamcinolone (TA)
Sharma et al. (2018) [52]	50	10 days	TA & Amlexanox are more efficacious in reducing ulcer size.	Amlexanox & triamcinolone were equally effective in patients suffering from RAS
Shrivasta et al. (2018) [53]	40	5 days	90% of TA participants showed completely healed ulcers in 70% of TA.	65% of amlexanox participants showed complete improvement of peri-ulcer erythema & 70% for pain reduction. Completely healed ulcers in 75% of participants in the amlexanox group.
Ofluoglu et al. (2017) [49]	180 patients	6 days	Effective but slower pain reduction than TGO	TGO showed greater pain reduction and faster ulcer healing
Koray et al. (2016) [47]	60	7 days	Provided pain relief but no significant difference in ulcer size and VAS scores on day 0	The pain score in the HA group was statistically lower than that of the TA groups on days 4 and 7
Deshmukh & Bagewadi (2014) [41]	60	7 days	No statistical significance	Curcumin & TA showed similar efficacy in the treatment of minor RAS. Curcumin gel can be used as an effective and safer alternative to steroids in the treatment of RAS.
El-Haddad et al. (2014) [45]	94 patients, 180 ulcers	5 days	Effective but slower healing than honey	Honey was significantly more effective in reducing ulcer size, pain, and erythema.

**Table 3:** Summary of natural, alternative, and combination therapies compared with triamcinolone for recurrent aphthous stomatitis.

Alternative or Combination Therapy	Studies, Arranged by Year	Sample Size	Follow-Up	Summary of Comparative Findings Versus Triamcinolone	Adverse Effects/Tolerability
Honey and honey-based therapies	Ghalwash et al. (2025); Mhapuskar et al. (2022); El-Haddad et al. (2014) [42,43,45]	30; 60; 94 patients	7 days; 7 days/until healing; 5 days	Thyme honey showed better pain relief and ulcer size reduction than TA. Honey improved pain, ulcer size, and healing and was superior to TA in the earlier studies.	No adverse effects were reported with honey
Curcumin	Kia et al. (2020); Raman et al. (2020); Deshmukh and Bagewadi (2014) [39,40,41]	58; 60; 60	10 days; 7 days and 6 months; 7 days	Curcumin demonstrated efficacy comparable to 0.1% TA for pain reduction and ulcer healing. It was considered a potential alternative where corticosteroid use is undesirable.	No adverse effects reported in the provided summaries.
Punica granatum	Lavaee et al. (2024); Tavangar et al. (2019) [26,36]	60; 60	6 days; 5 days	Both studies favored P. granatum, demonstrating greater reductions in pain and lesion size and faster healing than TA.	Not reported.
Sumac/Rhus coriaria	Rezvani et al. (2025); Lavaee et al. (2022) [34,35]	60; 22	7 days; 6 days	Sumac showed shorter healing time and improved lesion size and pain outcomes. In the earlier study, TA provided better pain reduction, whereas R. coriaria showed slightly better ulcer healing.	Not reported.
Aloe vera	Giroh et al. (2019) [38]	34	7 days	TA was superior for ulcer healing, whereas aloe vera provided better pain relief and reduction in burning sensation.	Not reported.
Honey-lemon spray	Shirzaiy et al. (2023) [44]	46	7 days	Similar efficacy to TA for the evaluated clinical outcomes.	Well tolerated.
Salvizan	Abbasi et al. (2023) [33]	60	8 days	Faster pain recovery and shorter ulcer healing time than TA.	Not reported.
HiOra SG gel	Nayak et al. (2023) [50]	50	10 days	Comparable efficacy to TA, with better tolerability.	Some side effects reported with TA; HiOra SG was better tolerated.
TGO	Ofluoglu et al. (2017) [49]	180	6 days	Greater pain reduction and faster ulcer healing than TA.	Not reported.
CBD	Umpreecha et al. (2023) [37]	69	7 days	CBD reduced ulcer size and accelerated healing. TA remained the preferred intervention, but CBD was considered a potential alternative for patients declining topical corticosteroids.	No side effects reported with CBD.
Hyaluronic acid	Mustafa and Alzubaidee (2020); Koray et al. (2016) [46,47]	80; 60	6 days; 7 days	HA generally provided greater pain relief and faster healing. In one study, ulcer size outcomes were similar, but pain scores favored HA at later follow-up.	Not reported.
Amlexanox	Sharma et al. (2018); Shrivasta et al. (2018) [52,53]	50; 40	10 days; 5 days	Amlexanox and TA demonstrated broadly comparable efficacy for ulcer healing and symptom reduction.	Not reported.
Ora-Aid	Barzegar et al. (2024) [51]	40	7 days	TA was superior for ulcer size reduction, whereas Ora-Aid was more effective for pain and burning sensation relief.	Not reported.
Laser therapy	Huo et al. (2021); Hussein et al. (2021); Kour and Dwivedi (2020); Seyyedi et al. (2020) [29,30] [31,32]	56; 28; 45; 15	Approximately 6–7 days	Laser-based therapies generally provided faster pain relief and ulcer healing than TA. CO_2_ laser showed superior pain relief but similar ulcer size reduction.	Not reported.
Hyaluronic acid + microbiome therapy	Ramesh and Sadaksharam (2021) [48]	45	7 days	The combination therapy resulted in faster healing and better pain relief than TA.	Not reported.

**Table 4:** Summary of management and treatment for RAS.

**Diagnostic Stage**	**Clinical Decision**	**Management**
Patient presents with oral ulcer(s)	Clinical evaluation	Begin diagnostic pathway
Confirm RAS diagnosis	Rule out systemic causes	If uncertain, evaluate Behçet’s disease, Crohn’s disease/UC, hematologic and immunologic disorders
Classify ulcer severity	Mild/Minor	Honey/Propolis; Sage gel; Curcumin; Aloe vera; Pomegranate extract
	Moderate	Hyaluronic acid gel; Mucoadhesive patches; TGO formulation; Amlexanox 5%; Chlorhexidine gel; Topical anesthetics
	Severe/Refractory	Triamcinolone 0.1%; Low-level laser therapy; Diode/CO_2_ laser; HA + microbiome therapy; Consider systemic therapy
Corticosteroid tolerance	Intolerant	Honey/Curcumin/Sage; Hyaluronic acid; Low-level laser therapy; TGO formulation
	Steroids acceptable	Triamcinolone acetonide 0.1%; Apply 2–4× daily; Short course (<2 weeks); Monitor candidiasis
Reassessment	7–14 days	Evaluate healing
Outcome	Not healing/recurrent	Add LLLT or laser therapy; HA + microbiome; Investigate triggers; Oral medicine referral
Outcome	Healed/resolving	Nutritional support (B12, Fe, Zn); Continue therapy; Follow-up

## Data Availability

The data supporting the findings of this study are available from the corresponding author upon reasonable request.

## Supplementary Materials

The following supporting information can be downloaded at: https://www.mdpi.com/article/10.3390/biomedicines14102260/s1, Supplementary Material S1. PRISMA Checklist.

## Abbreviations

The following abbreviations are used in this manuscript:

CBD	Cannabidiol
CIs	Confidence intervals
HA	Hyaluronic acid
LLLT	Low-level laser therapy
OAUs	Oral aphthous ulcers
PRISMA	Preferred Reporting Items for Systematic Reviews and Meta-Analyses
RCTs	Randomized controlled trials
RAS	Recurrent aphthous stomatitis
SMD	Standardized mean difference
TA	Triamcinolone
TGO	Triester glycerol oxide
VAS	Visual analogue scale

## References

[1] Scully C., Porter S. (2008). Oral mucosal disease: Recurrent aphthous stomatitis. Br. J. Oral Maxillofac. Surg..

[2] Giannetti L., Murri Dello Diago A., Lo Muzio L. (2018). Recurrent aphthous stomatitis. Minerva Stomatol..

[3] Edgar N.R., Saleh D., Miller R.A. (2017). Recurrent aphthous stomatitis: A review. J. Clin. Aesthet. Dermatol..

[4] Lehner T. (1969). Pathology of recurrent oral ulceration and oral ulceration in Behçet’s syndrome: Light, electron and fluorescence microscopy. J. Pathol..

[5] Akintoye S.O., Greenberg M.S. (2014). Recurrent aphthous stomatitis. Dent. Clin. N. Am..

[6] Laskaris G. (2023). Aphthous stomatitis. European Handbook of Dermatological Treatment.

[7] Staines K., Greenwood M. (2015). Aphthous ulcers (recurrent). BMJ Clin. Evid..

[8] Altenburg A., El-Haj N., Micheli C., Puttkammer M., Abdel-Naser M.B., Zouboulis C.C. (2014). The treatment of chronic recurrent oral aphthous ulcers. Dtsch. Arztebl. Int..

[9] Khandwala A., Van Inwegen R.G., Alfano M.C. (1997). 5% amlexanox oral paste, a new treatment for recurrent minor aphthous ulcers: I. Clinical demonstration of acceleration of healing and resolution of pain. Oral Surg. Oral Med. Oral Pathol. Oral Radiol. Endod..

[10] Liu H., Tan L., Fu G., Chen L., Tan H. (2022). Efficacy of topical intervention for recurrent aphthous stomatitis: A network meta-analysis. Medicina.

[11] McBride D.R. (2000). Management of aphthous ulcers. Am. Fam. Physician.

[12] Akerzoul N., Chbicheb S. (2018). Low laser therapy as an effective treatment of recurrent aphthous ulcers: A clinical case reporting two locations. Pan Afr. Med. J..

[13] Piacentini M., Borghetti R.L., Zancanaro de Figueiredo M.A., Cherubini K., Gonçalves Salum F. (2019). Doxycycline: An option in the treatment of ulcerated oral lesions?. J. Clin. Pharm. Ther..

[14] Cheng B., Zeng X., Liu S., Zou J., Wang Y. (2020). The efficacy of probiotics in management of recurrent aphthous stomatitis: A systematic review and meta-analysis. Sci. Rep..

[15] Al-Omiri M.K., Alhijawi M., Alzarea B.K., Hassan R.S.A., Lynch E. (2016). Ozone treatment of recurrent aphthous stomatitis: A double blinded study. Sci. Rep..

[16] El-Wakeel N.M., Dawoud M.H.S. (2019). Topical insulin-liposomal formulation in management of recurrent aphthous ulcers: A randomized placebo-controlled trial. J. Investig. Clin. Dent..

[17] Brookes Z.L.S., Bescos R., Belfield L.A., Ali K., Roberts A. (2020). Current uses of chlorhexidine for management of oral disease: A narrative review. J. Dent..

[18] Savadori P., Rai P.M., Tadakamadla S., Khijmatgar S., Inchingolo F., Greco C., Occhipinti C., Fahim M.S., Del Fabbro M., Tartaglia G.M. (2022). Minor recurrent aphthous ulcer management with hyaluronic acid gel in an Italian cohort: A double-blind randomized clinical trial. BioMed Res. Int..

[19] Zou H., Liu Z., Wang Z., Fang J. (2022). Effects of *Aloe vera* in the treatment of oral ulcers: A systematic review and meta-analysis of randomized controlled trials. Oral Health Prev. Dent..

[20] Seyyedi S.A., Sanatkhani M., Pakfetrat A., Olyaee P. (2014). The therapeutic effects of chamomilla tincture mouthwash on oral aphthae: A randomized clinical trial. J. Clin. Exp. Dent..

[21] Dorsareh F., Vahid-Dastjerdi G., Bouyahya A., Zarshenas M.M., Rezaie M., Yang W.M., Amiri-Ardekani E. (2023). Topical licorice for aphthous: A systematic review of clinical trials. Iran. J. Med. Sci..

[22] Nambiar K.R., Krupashankar R., Veena S.N., Kavitha A.P. (2021). Comparison of virgin coconut oil with 5% amlexanox in management of minor recurrent aphthous ulcers: A randomized control trial. RGUHS J. Dent. Sci..

[23] Roberts T., Kallon I.I., Schoonees A. (2024). Efficacy and safety of propolis for treating recurrent aphthous stomatitis (RAS): A systematic review and meta-analysis. Dent. J..

[24] Elamrousy W., Mortada A., Shoukheba M. (2021). Evaluation of novel topical camel whey protein gel for the treatment of recurrent aphthous stomatitis: Randomized clinical study. J. Int. Soc. Prev. Community Dent..

[25] Ghalayani P., Zolfaghary B., Farhad A.R., Tavangar A., Soleymani B. (2013). The efficacy of *Punica granatum* extract in the management of recurrent aphthous stomatitis. J. Res. Pharm. Pract..

[26] Tavangar A., Aslani A., Nikbakht N. (2019). Comparative study of *Punica granatum* gel and Triadent oral paste effect on recurrent aphthous stomatitis, a double blind clinical trial. J. Dent..

[27] Moher D., Liberati A., Tetzlaff J., Altman D.G., PRISMA Group (2010). Preferred reporting items for systematic reviews and meta-analyses: The PRISMA statement. Int. J. Surg..

[28] Fekete J.T., Győrffy B. (2025). MetaAnalysisOnline.com: Web-based tool for the rapid meta-analysis of clinical and epidemiological studies. J. Med. Internet Res..

[29] Huo X., Han N., Liu L. (2021). Effect of different treatments on recurrent aphthous stomatitis: Laser versus medication. Lasers Med. Sci..

[30] Hussein H., Zaky A., Nadim M., Elbarbary A. (2021). Low-level diode laser therapy (LLLT) versus topical corticosteroids in the management of recurrent aphthous stomatitis patients: A randomized controlled trial. Adv. Dent. J..

[31] Kour M., Dwivedi A. (2020). A comparative randomized clinical trial to assess the effectiveness of diode lasers in both contact and non-contact mode and 0.1% triamcinolone acetonide mouth paste in treatment of oral ulcers—An in vivo study. Lasers Dent. Sci..

[32] Seyyedi S.A., Olyaee P., Fekrazad R., Partovi S., Baghizadeh Fini M. (2020). The effect of carbon dioxide laser on aphthous stomatitis treatment: A double-blind randomized clinical trial. J. Lasers Med. Sci..

[33] Abbasi F., Rasoulzadeh Z., Yavari A. (2023). The effect of sage (Salvizan gel) compared to triamcinolone acetonide on the treatment of recurrent aphthous stomatitis: A double-blinded randomized clinical trial. BMC Oral Health.

[34] Lavaee F., Ghasemi M., Amiri M.A., Farshidfar N. (2022). The comparative effects of *Rhus coriaria* and triamcinolone in patients with recurrent aphthous stomatitis: A single-blinded randomized controlled clinical trial. BioMed Res. Int..

[35] Rezvani Z., Cheshme Soltani M.T., Mirzadeh M., Gheibi N., Mokhlesi A., Zarabadipour M. (2025). Comparison of two oral pastes containing triamcinolone and *Rhus coriaria* L. for treating minor aphthous lesions. Int. Dent. J..

[36] Lavaee F., Parhizkar E., Tavakoli F., Zarshenas M.M., Asmarian N. (2024). Evaluation of the effects of *Punica granatum* flower tablets on pain and healing of minor recurrent aphthous stomatitis: A randomized clinical trial. Clin. Exp. Dent. Res..

[37] Umpreecha C., Bhalang K., Charnvanich D., Luckanagul J. (2023). Efficacy and safety of topical 0.1% cannabidiol for managing recurrent aphthous ulcers: A randomized controlled trial. BMC Complement. Med. Ther..

[38] Giroh V., Hebbale M., Mhapuskar A., Hiremutt D., Agarwal P. (2019). Efficacy of aloe vera and triamcinolone acetonide 0.1% in recurrent aphthous stomatitis: A preliminary comparative study. J. Indian Acad. Oral Med. Radiol..

[39] Raman P., Pitty R., Krithika C.L., Anand S.P.N., Subramani G.P. (2020). Topical curcumin and triamcinolone acetonide in recurrent minor aphthous ulcers: A pilot trial. J. Contemp. Dent. Pract..

[40] Kia S.J., Mansourian A., Basirat M., Akhavan M., Mohtasham-Amiri Z., Moosavi M.S. (2020). New concentration of curcumin orabase in recurrent aphthous stomatitis: A randomized, controlled clinical trial. J. Herb. Med..

[41] Deshmukh R., Bagewadi A. (2014). Comparison of effectiveness of curcumin with triamcinolone acetonide in the gel form in treatment of minor recurrent aphthous stomatitis: A randomized clinical trial. Int. J. Pharm. Investig..

[42] Ghalwash D., El-Gawish A., Ammar A., Abou-Bakr A. (2025). Efficacy of thyme honey in the management of oral aphthous ulcers: A randomized controlled clinical trial. Eur. J. Oral Sci..

[43] Mhapuskar A.A., Thakare S., Kale I.P., Karmarkar P., Hiremutt D.R.P., Jadhav A. (2022). Comparison of effects of honey and 0.1% triamcinolone acetonide in the management of recurrent aphthous stomatitis: A randomized, single-blind study. J. Indian Acad. Oral Med. Radiol..

[44] Shirzaiy M., Heydarirad G., Bakhshipour S. (2023). Effects of honey-lemon spray on recurrent aphthous stomatitis compared to triamcinolone ointment. Braz. J. Oral Sci..

[45] El-Haddad S.A., Asiri F.Y., Al-Qahtani H.H., Al-Ghmlas A.S. (2014). Efficacy of honey in comparison to topical corticosteroid for treatment of recurrent minor aphthous ulceration: A randomized, blind, controlled, parallel, double-center clinical trial. Quintessence Int..

[46] Mustafa B.N., Fakhree Alzubaidee A. (2020). Comparative evaluation of the efficacy of topical hyaluronic acid (0.2%) and topical triamcinolone acetonide (0.1%) in the treatment of recurrent aphthous stomatitis. Diyala J. Med..

[47] Koray M., Ofluoglu D., A S., Issever H., Yaltirik M. (2016). The efficacy of hyaluronic acid gel in pain control of recurrent aphthous stomatitis. Int. J. Dent. Oral Sci..

[48] Kuduva Ramesh S.S., Sadaksharam J. (2021). Assessment of clinical efficacy of microbiome and topical hyaluronic acid gel combination therapy in minor aphthous ulcer management: A comparative study. J. Indian Acad. Oral Med. Radiol..

[49] Ofluoglu D., Ergun S., Warnakulasuriya S., Namdar-Pekiner F., Tanyeri H. (2017). An evaluation of the efficacy of a topical gel with Triester Glycerol Oxide (TGO) in the treatment of minor recurrent aphthous stomatitis in a Turkish cohort: A randomized, double-blind, placebo-controlled clinical trial. Med. Oral Patol. Oral Cir. Bucal.

[50] Nayak N.G., Modi P., Shah S., Patel P., Patel D.D., Patel R.R., Hapani D. (2023). Comparison of effectiveness of Hiora SG gel with triamcinolone acetonide gel in recurrent aphthous stomatitis. Cureus.

[51] Barzegar M., Ghadirian H., Roustaeizade Shooroki Z., Yektaie M.A., Pouyafard A. (2024). Comparative efficacy of Triadent and Ora-Aid mucoadhesive patch for treatment of minor aphthous ulcers: A clinical trial. J. Res. Dent. Maxillofac. Sci..

[52] Sharma R., Pallagatti S., Aggarwal A., Sheikh S., Singh R., Gupta D. (2018). A randomized, double-blind, placebo-controlled trial on clinical efficacy of topical agents in reducing pain and frequency of recurrent aphthous ulcers. Open Dent. J..

[53] Shrivastava K., Naidu G., Deshpande A., Handa H., Raghuvanshi V., Gupta M. (2018). Comparative evaluation of the efficacy of topical amlexanox 5% oral paste and triamcinolone acetonide 0.1% oral paste in the treatment of recurrent aphthous stomatitis (RAS). J. Indian Acad. Oral Med. Radiol..

[54] Yasir M., Goyal A., Sonthalia S. (2025). Corticosteroid adverse effects. StatPearls.

[55] D’Amario M., Foffo G., Grilli F., Capogreco M., Pizzolante T., Rastelli S. (2025). Treatments for recurrent aphthous stomatitis: A literature review. Dent. J..

[56] Shavakhi M., Sahebkar A., Shirban F., Bagherniya M. (2022). The efficacy of herbal medicine in the treatment of recurrent aphthous stomatitis: A systematic review of randomized clinical trials. Phytother. Res..

[57] Li C.L., Huang H.L., Hua H., Wang W.C. (2015). Efficacy and safety of topical herbal medicine treatment on recurrent aphthous stomatitis: A systematic review. Drug Des. Dev. Ther..

[58] Amanpour S., Akbari Javar M., Sarhadinejad Z., Doustmohammadi M., Moghadari M., Sarhadynejad Z. (2023). A systematic review of medicinal plants and herbal products’ effectiveness in oral health and dental cure with health promotion approach. J. Educ. Health Promot..

[59] Parra-Moreno F.J., Egido-Moreno S., Schemel-Suárez M., González-Navarro B., Estrugo-Devesa A., López-López J. (2023). Treatment of recurrent aphthous stomatitis: A systematic review. Med. Oral Patol. Oral Cir. Bucal.

[60] Al-Maweri S.A., Alaizari N., Alanazi R.H., Ashraf S., Alanazi R.H., Al-Sharani H.M., Halboub E. (2021). Efficacy of hyaluronic acid for recurrent aphthous stomatitis: A systematic review of clinical trials. Clin. Oral Investig..

[61] Abidi A.H., Alghamdi S.S., Derefinko K. (2022). A critical review of cannabis in medicine and dentistry: A look back and the path forward. Clin. Exp. Dent. Res..

[62] Suter V.G.A., Sjölund S., Bornstein M.M. (2017). Effect of laser on pain relief and wound healing of recurrent aphthous stomatitis: A systematic review. Lasers Med. Sci..

[63] Lalabonova H., Daskalov H. (2014). Clinical assessment of the therapeutic effect of low-level laser therapy on chronic recurrent aphthous stomatitis. Biotechnol. Biotechnol. Equip..

[64] Shalaby H. (2019). Effect of diode laser combined with hyaluronic acid gel in treatment of recurrent aphthous stomatitis in children: Randomized clinical trial. Egypt. Dent. J..

